# Evaluating the Fidelity of *De Novo* Short Read Metagenomic Assembly Using Simulated Data

**DOI:** 10.1371/journal.pone.0019984

**Published:** 2011-05-23

**Authors:** Miguel Pignatelli, Andrés Moya

**Affiliations:** 1 Unitat Mixta d'Investigació en Genòmica i Salut, Centre Superior d'Investigació en Salut Pública/UVEG-Institut Cavanilles, Valencia, Spain; 2 CIBER en Epidemiología y Salud Pública (CIBERESP), Barcelona, Spain; 3 European Bioinformatics Institute, Wellcome Trust Genome Campus, Hinxton, United Kingdom; J. Craig Venter Institute, United States of America

## Abstract

A frequent step in metagenomic data analysis comprises the assembly of the sequenced reads. Many assembly tools have been published in the last years targeting data coming from next-generation sequencing (NGS) technologies but these assemblers have not been designed for or tested in multi-genome scenarios that characterize metagenomic studies. Here we provide a critical assessment of current *de novo* short reads assembly tools in multi-genome scenarios using complex simulated metagenomic data. With this approach we tested the fidelity of different assemblers in metagenomic studies demonstrating that even under the simplest compositions the number of chimeric contigs involving different species is noticeable. We further showed that the assembly process reduces the accuracy of the functional classification of the metagenomic data and that these errors can be overcome raising the coverage of the studied metagenome. The results presented here highlight the particular difficulties that *de novo* genome assemblers face in multi-genome scenarios demonstrating that these difficulties, that often compromise the functional classification of the analyzed data, can be overcome with a high sequencing effort.

## Introduction

Metagenomics is an emergent field aimed at studying the genomic material recovered directly from samples either environmental or from living beings. Its main goal is to provide a detailed view of the organism composition and functional properties at different levels of the communities, particularly bacterial ones, under study. Many microbial communities from different environments have been studied during the last decades using these techniques [1], [2]. Recent development of high parallel sequencing technologies has provoked a profound impact in this field and has put metagenomic experiments within the range of many microbiological laboratories in terms of budget, time and work. The classic 16S rRNA surveys to quantify microbial diversity has given way to metagenomic studies where the full genomic content of the communities is sequenced to obtain the bacterial composition and functional repertoire present in the environment of interest. Because of this expansion of metagenomic research many tools to facilitate the taxonomical and functional classification of these experiments have been developed in recent years (see for example, [2], [3], [4], [5], [6], [7], [8], [9], [10], [11], [12] and the review in [13]).

The catalog of *de novo* genome assembly algorithms has been adapted and expanded with the advent of the so-called next generation sequencing (NGS) platforms. The higher amount of DNA obtained, the shorter length of the produced reads, the higher error rates in the sequences obtained compared with the classical Sanger method and the particular characteristics of those errors have prevented an easy adaptation of classic assembly algorithms to work with NGS data (for a comprehensive review see [14] and [15]). Almost all the assembly tools developed so far use variations of three fundamental assembly strategies. The greedy algorithm used by CAP3 [16], Phrap [17] and TIGR assembler [18] is conceptually the simplest solution to genome assembly and new tools tailored to NGS data have been developed recently like SSAKE [19], SHARCGS [20] or VCAKE [21]. But maybe the most popular algorithmic solution is the Overlap-Layout-Consensus (OLC) algorithm used in the Celera Assembler [22], Arachne [23], [24], PCAP [25] or Mira to name a few. With the consolidation of the NGS platforms, new tools based on this algorithm have also emerged like Newbler, Minimus [26] or Edena [27]. More recently, new strategies based on Eulerian paths (and in particular, deBruijn graphs) have become popular hampered by the high computational demanding imposed by the NGS data. The most notable examples are Velvet [28], Euler [29], SOAPdenovo [30], ABySS [31] and ALLPATHS [32].

All the abovementioned software targets the assembly of single genomes where the fundamental problem is the presence of repeated DNA fragments in the target sequence. This problem is far from trivial and converts the assembly problem in unsolvable without additional data like mate pair information. These computational difficulties have lead to the adoption of many different heuristic assemblers that convert them in very specialized tools for the tasks they are conceived (the assembly of individual genomes) preventing an easy or direct adaptation to different scenarios like metagenomic or cDNA analysis.

Although it has been shown that it is possible to reconstruct almost complete genomes from very simple metagenomic samples [33] the rationale behind metagenome assembly is to obtain contigs to boost the accuracy of their functional and taxonomical classification. But metagenome assembly has to face particular difficulties, such as: *i*) the co-existence of related species and multiple strains of the same species; *ii*) the particular restrictions of the genome-oriented assemblers, for example uniform coverage is usually expected by most of the assembly tools; *iii*) horizontal gene transfer (HGT) events between co-existence species or *iv*) the high diversity of starting genomic material to sequence that requires a high sequencing effort. Despite these difficulties metagenomic data is often assembled to improve its annotation (see for example [34], [35]) but the trade-off between the noise of the resulting contigs (specially when short reads are used) and the gain in sequence length has not been attended enough.

Recently, Mavromatis et al have studied the problem of metagenomic assembly using simulated datasets of Sanger reads [36]. In the present study we address the problem of *de novo* short read metagenome assembly using simulated data to provide a comprehensive assessment of the current assembly technologies and how this process affects the functional classification of the assembled contigs.

## Results

### Simulations

In Mavromatis et al, Sanger reads from different genomes were mixed to form three different simulated metagenomics datasets of different complexity (low, medium and high, named as LC, MC and HC respectively). In the LC dataset, a sizeable portion of the reads belongs to a dominant organism, the MC dataset has a few dominant organisms (some of them taxonomically related) and in the HC dataset no dominant organism is present in the mix. These datasets were used to assess the fidelity of different tools commonly used for metagenomics analysis [36]. We have adapted these artificial metagenomes to the typical length of current next-generation sequencing technologies. The genomes present in our simulated microbiomes were selected by picking up the same species described in Mavromatis et al from the set of complete genomes available at the NCBI repository. When one particular strain was not found, we picked up a close relative (usually a different strain). From these genomes we randomly sampled DNA fragments maintaining the same genome coverage specified in Mavromatis et al, but adapting the number of reads and their length to meet the characteristics of current 454 and Illumina technologies (400 bp and 110 bp, respectively). It is important to note that the number of sequences and the taxonomical distribution of these datasets (LC, MC and HC) are almost identical and what really differs them is the relative abundance of each organism in each simulated community (Table 1 and Figure S1). To evaluate the sequencing effort in metagenome assembly we also re-sampled the HC dataset with the coverage of each genome ten times higher than in the original dataset (HChc dataset). A total of 3,270,435 400 bp and 11,891,463 110 bp fragments were generated for approximately 1,3 Gb of total sequence of each type. All this sampling information is summarized in Table 1 and the individual composition of each dataset is presented in Dataset S1.

**Table 1:** Summary of the simulated and real datasets used in this study.

			Number of reads	
Dataset	Number of species	Number of base pairs	400 bp	110 bp
LC	112	88 Mb	220288	801062
MC	110	107 Mb	269583	980312
HC	113	101 Mb	252754	919099
HC-hc	113	1,01 Gb	2527540	9190990
Oral	?	203 Mb	464594*	-

*Mean length of reads of 438 bp.

It is well known that one of the higher drawbacks of 454 and Illumina technologies is their high rate of sequencing errors compared to the Sanger technology [37], [38]. In addition, the kind of errors committed is characteristic of each technology. For 454 reads, problems in the determination of homopolymer lengths as well as other more subtle biases (like *carry forward* and *incomplete extension* events) have been described [37]. Some of these errors (in particular, homopolymer length determination problems) can be modeled and simulated *in silico* [38]. In real datasets, though, it has been observed that more than 80% of the reads are error-free, with most of the errors accumulating in the remaining 20% [39]. The error rate of the Illumina platform has been described to be around 0.5–1% over the entire read, most of them being substitution errors with a low number of insertions and deletions [40]. The frequency of errors in Illumina reads is position dependent and most of them accumulating at the 3′ end of the reads (>3% of errors). These kinds of errors have been previously modeled following a fourth degree polynomial [41]. We applied these errors models to our datasets (see methods) to test the assemblers both in error-free simulations and with datasets containing typical NGS errors.

### Assembly

The metagenomes were assembled using different *de novo* short read genomic assemblers. For the 400 bp simulations Newbler (the “official” 454 assembler from Roche) and Celera Assembler [22] were used while for the 110 bp simulations we used SSAKE [19] and Velvet [28]. Newbler is probably the most popular assembler for 454 data, while the Celera Assembler has been used in big genomic [22], [42] and metagenomic projects [43], [44] and has been recently adapted to work with 454 sequences [45]. Both are based on the OLC strategy. SSAKE follows a greedy algorithm and has been also used to assembly metagenomic sequences while Velvet is one of the most popular deBruijn based assemblers. All these assemblers were run with options that allowed the traceability of each read in the final contigs. This strategy allowed us to identify and quantify misplaced reads in the final set of contigs. Basic statistics for these assemblies are summarized in Tables 2 and S1. Our results show that, as expected, the most affecting variables in the assembly process are the complexity of the metagenome and the read length (although the coverage between 110 bp and 400 bp datasets is the same). We compared this result with the assembly of a real 454 oral microbiota dataset and found that both the N50 and the length of the longest contigs are in accordance with the assemblies of simulated data. This real population can be considered of low complexity because it is dominated by a rather small number of organisms that are highly represented (Belda P. et al, under review). In all cases, the introduction of typical sequencing errors had a negative impact in the assembly process (Table S1). Interestingly, Newbler seems to deal particularly well with 454 homopolymeric length determination errors. For Illumina datasets, the introduction of positional dependent errors has a similar negative impact in both assemblers used.

**Table 2:** Summary of the assembly statistics of the simulated datasets.

	Assembler	LC			MC			HC			HChc		
		N50(bps)	Longest contig (bp)	% of reads in chimeras	N50(bps)	Longest contig (bp)	% of reads in chimeras	N50(bps)	Longest contig (bp)	% of reads in chimeras	N50(bps)	Longest contig (bp)	% of reads in chimeras
**400 bp**	**Newbler**	3685	31468	3.88	1883	23915	9.75	608	2848	12.57	1433	39814	5.74
	**Celera**	5700	48060	1.65	1978	16971	4.71	588	3038	11.85	1676	46528	3.11
**110 bp**	**SSAKE**	190	2011	0.22	181	4193	2.33	128	1822	6.02	129	6313	3.02
	**Velvet**	181	3019	4.11	170	4210	7.15	141	2201	8.34	182	5925	5.49

Only contigs longer than the read size were considered.

### Taxonomical analysis of contigs

The resulting contigs were assigned to the organism that contributed the majority of its constituent reads. We then calculated the proportion of reads miss-assembled in contigs assigned to another organism. As can be seen in Table 2, in the error-free datasests, this proportion increases notably with the complexity of the metagenome, ranging from 0.22% (LC assembled with SSAKE) to 12.57% (HC assembled with Newbler). Interestingly, we have not found differences in chimericity between 400 bp and 110 bp assemblies. These results are not affected by changes in basic parameters of the assemblers like the kmer length in SSAKE and VELVET and the minimum percentage identity for unitigs in Celera and Newbler as explained in *Methods* (data not shown). The assemblies using datasets with sequencing errors can be considered worse based on N50 and length of the longest contig, but, interestingly, only a modest increase in chimericity is observe, suggesting that reads with errors are more likely to be left out by the assembler instead of being used and misplaced in chimeric contigs (Table S1).

**Figure 1:**
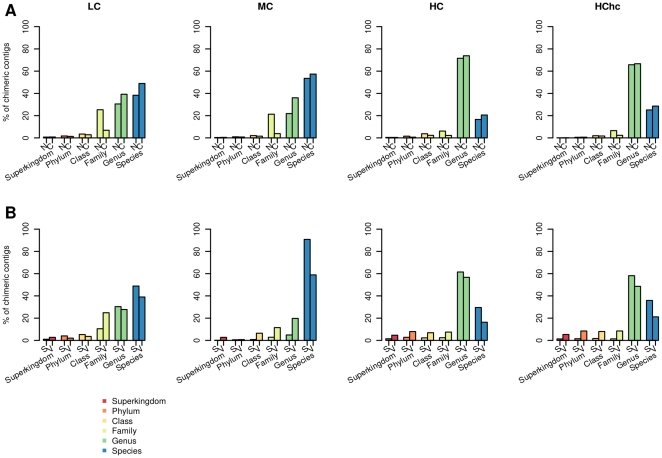
Taxonomic level of the lowest common ancestor of the chimeric contigs. (A) 400 bp and (B) 110 bp datasets respectively. *N* stands for Newbler, *C* for Celera Assembler, *S* for SSAKE and *V* for Velvet.

We also calculated for each chimeric contig the taxonomic lower common ancestor (LCA) of their reads. As can be seen in Figure 1, most of the chimeric contigs formed by the Celera Assembler and SSAKE are composed by species belonging to the same genus or species while chimeric contigs formed by Newbler and Velvet are composed by species belonging the the same family or a deeper taxonomic rank. It is also noteworthy that for the LC and MC datasets a sizeable number of chimeric contigs were composed by organisms belonging to the same species or strain. This is true regardless the length of the fragments (400 bp and 110 bp). For the HC dataset, though, the taxonomic relationship of most of reads forming chimeric contigs raises to the genus level, even when a high coverage sampling was used (HChc dataset). This result shows the inherent difficulty of assembling complex metagenomic populations even when the sequence space of the population is exhausted. Similar results were obtained when the datasets with induced errors were used (Figure S2) suggesting that sequencing errors may have a small effect in the formation of trans-chimeric contigs.

As can be seen in Figure S3 not all the taxonomically related organisms are equally presented in the chimeric contigs. There seem to be “hot spots” or groups of organisms that are the major contributors to chimericity. Also, genomic coverage seems not to be a relevant factor in the definition of these “hot spots” since they are formed by high coverage organisms but also by low or medium coverage organisms as well. The same “hot spots” covering the same species can be reproduced when the clustering is done based on whole-genomic sequence similarity instead of taxonomic relationship (Figure S4), suggesting that sequence similarity is the main cause of contig chimericity.

### Sequence divergence

**Figure 2:**
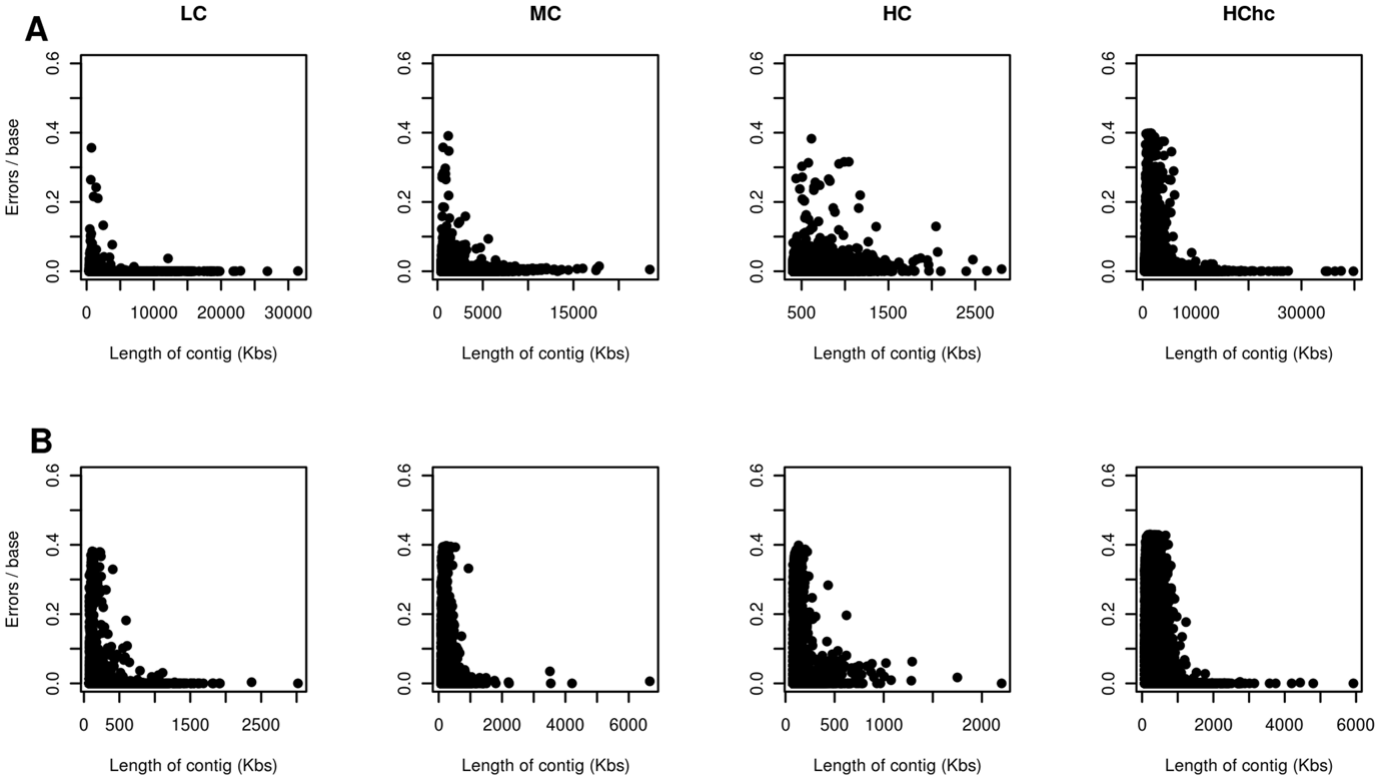
Sequence divergence degree of contigs with respect to the reference genomes. (A) Newbler (400 bp datasets) and (B) Velvet (110 bp datasets) assemblies.

The presence of miss-assembled reads in a contig doesn't necessarily mean a significant divergence between the contig and the reference sequence. To see to what extend miss-assembled reads distort the consensus sequence of chimeric contigs with respect to the original reference we compared all the contigs with the genomes used for sampling and for the best hit, we calculated their sequence divergence. In Figure 2, the number of errors per base for each contig obtained with the Newbler (400 bp samples) (Figure 2A) and Velvet (110 bp samples) (Figure 2B) assemblers is plotted against the contig length showing that most of the errors accumulate on short contigs. Again, this is highly dependent on metagenome complexity, with the HC dataset having more errors in its longer contigs.

### Functional analysis

**Figure 3:**
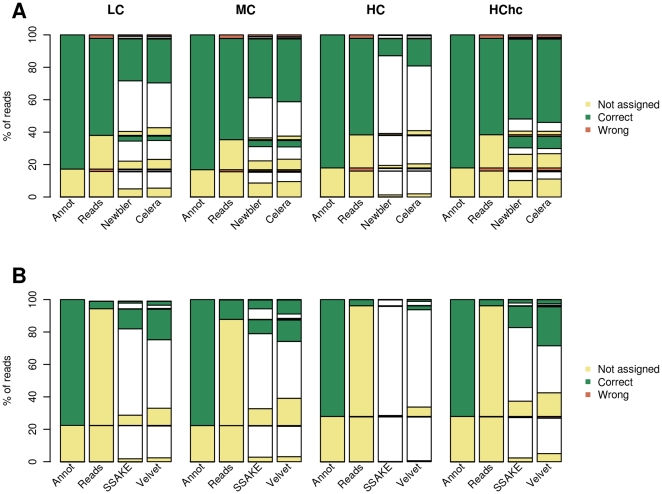
Functional annotation of the reads at different levels. (A) 400 bp and (B) 110 bp datasets respectively. The first column of each group differentiates between reads lacking (yellow) or having (green) a real functional annotation in the genome (see Methods). For each of these two categories, the second column differentiates between reads that lack (yellow) annotation or that have correct (green) or incorrect (red) annotation at the read level. For these categories, the third and fourth column differentiates between reads having correct (green), incorrect (red) or no (yellow) annotation at the contig level. Reads that are no present in the contigs are represented in the white boxes.

In metagenomics, it is widely assumed that longer contigs also permits a better functional classification than the individual reads, but the noise accumulated in chimeric contigs may act in the opposite direction hiding real homologies and lowering the sensitivity of homology searches. To study the impact of the assembly process on functional classification of metagenomic data we annotated the sampled “reads” and the assembled contigs using the COG database [46] as described in *Methods*. For each read we compared *i*) its COG classification reported in its genome of origin (we call this the “real annotation”), *ii*) its COG classification using the read sequences as BLAST input (the annotation at the read level) and *iii*) its COG classification using the contig sequences as BLAST input and inferring their annotation from their coordinates in the contig (the annotation at the contig level) (see Methods). Following this approach, for each read we compared the annotation of each fragment derived from the genomic sequence, its annotation using the read sequence itself and its annotation as being part of a contig. As can be seen in Figure 3, in accordance to the assembly goal, a significant set of reads can only be correctly annotated at the contig level and not at the read level. There is, however, another set of reads that are correctly annotated at the read level but can not be annotated at the contig level probably due to assembly errors that may be hiding real homologies.

For the 400 bp datasets it is surprising that the proportion of reads that lose their annotation due to the assembly process is similar (if not higher) than the proportion of reads that benefits from the assembly process to be annotated. For instance, for the MC dataset, the assembly with Newbler allow to annotate 10,672 reads that cannot be annotated at the read level, while 3,510 reads that can be correctly classified at the read level, loses their annotation at the contig level. For the HC dataset, the number of reads that at the contig level lost their correct annotation is much higher than the number of reads that benefits from the assembly process to get their correct annotation. Interestingly, the increase in coverage solves this problem as can be seen in the annotation of the HChc dataset where most of the reads are correctly annotated at the contig level.

When errors in the sequences were induced we didn't observe a significant increase in the proportion of mis-annotated reads (neither at the read nor at the contig level). The number of correctly annotated reads is slightly decreased both at the read level and at the contig level, while the number of unassembled reads is moderately increased. This result suggests that sequencing errors affects more dramatically the assembly process than the downstream functional classification of the contigs and this effect is largely alleviated by the increase in coverage (Figure S5).

The majority of miss-annotated reads are in chimeric contigs (71%) and these have a higher degree of chimericity (18%) than correctly annotated contigs (0.4%). This result confirms that contig chimericity is the main factor for miss-annotation of contigs. From these results it also follows that there are also some miss-annotated contigs that are not chimeric suggesting that other factors may be contributing to this effect, for example miss-assemblies where all the reads come from the same genome. Contigs that lead to miss-annotation have similar mean length (3,216 bp) than contigs correctly annotated (4,712 bp).

As for the 110 bp datasets, the percentage of reads annotated at the read level is very low (for the HC dataset, only 52,317 out of 919,099 110 bp reads can be assigned to a COG category). This makes that for all cases the annotation is always improved by the assembly process. This improvement decreases with the complexity of the metagenome, while the increase in coverage helps substantially in the annotation of the reads at the contig level. In particular, when the HChc dataset is assembled with SSAKE, only 1,911 reads (out of 9,190,990) are miss-annotated, while 1,932,014 reads not annotated at the read level are correctly annotated at the contig level.

The incorporation of errors to the 110 bp datasets has a similar impact than that observed for the 400 bp datasets with the number of correctly annotated reads at the read and contig levels being lowered.

**Figure 4:**
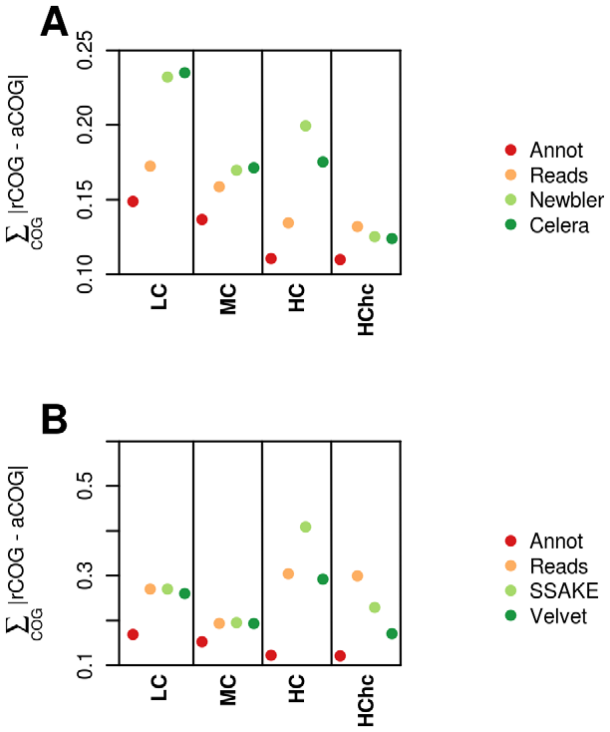
Global functional analysis. (A) 400 bp and (B) 110 bp datasets respectively. For each dataset, the COG category distribution of the genomes used for sampling was compared with the distribution of COGs categories obtained with the real (red) annotations and the annotations at the read (yellow) and contig (green and blue) levels. This comparisons are defined as the summatory of the differences of each COG category.

We next investigated if the functional assignment of these datasets accurately represents the functional content of the genomes of origin. To achieve this we represented the deviation between the functional distribution obtained for samples (based on the current annotation of the genomes), reads and contigs (based on BLAST homologies against the COG database) with the functional content of these entire genomes (Figure 4). In this figure, the nearer the points are to the X axis (lower Y values), the closer the tested functional distribution is from the functional distribution of the genomes of origin. We observed that for the 400 bp datasets (Figure 4A), the functional analysis at the read level (yellow dots) represents more accurately the COG distribution of the genomes sampled (red dots) than the assembled data (green and blue dots) except for the HChc dataset, where the fidelity of the annotation is slightly higher for the assemblies than for the set of individual reads. Interestingly, the oversampling doesn't contribute to a better annotation at the read level (yellow dots in HC and HChc), but it does affect dramatically the annotation of the assembled contigs (green and blue dots).

As expected given the small percentage of reads that are assembled or can be assigned to any functional category we obtain higher divergences for the 110 bp datasets (Figure 4B). For the LC and MC datasets the annotation at the read and contig level (yellow, green and blue dots) are similar to the annotation of the samples (red dots). For the HC dataset, though, there are substantial differences between the BLAST annotations (reads and contigs) and the annotation of the samples. As with the 400 bp datasets, oversampling does not affect the annotations at the read level, although it has a dramatic effect in the annotation of the contigs as can be seen in the HChc dataset where the overall COG annotation of contigs is far more precise than the annotation at the read level.

When the datasets containing errors where analyzed (Figure S6) a slightly higher discrepancy between the annotations and the real distribution of COGs is observed.

All these results suggest that metagenome assembly is in some cases of little help or even counterproductive in functional annotation and that the sequencing effort may be crucial when facing this kind of projects.

## Discussion

The field of metagenomics is reaching important milestones thanks to the new sequencing technologies appeared in the last years. Specific tools and algorithms designed to aid in the functional and taxonomical description of many different microbiomes have been actively developed during the last years [2], [6], [8], [10]. The 454 platform from Roche is being extensively used in the characterization of many microbial communities (see for example [47], [48], [49]) and more recently Qin et al have published the most comprehensive resource to date of the human gut metagenome from 124 individuals using the Illumina platform [34]. Unfortunately metagenome assembly still relies largely on tools targeting single microbial projects. As a result, metagenomic sequences are often subject to further analysis as a collection of short reads [13]. The only attempt to develop specific strategies to deal with metagenomic data we are aware of is the MetaORFA pipeline that relies on the EULER assembler [50] and the very recently published Genovo tool [51] based on a probabilistic model of read generation. Also, at the time of writing of this manuscript we had notice of the development of a still unpublished modified version of the Velvet assembler specially designed to deal with metagenomic sequences (MetaVelvet, Namiki T et al, unpublished). Not only the development of new algorithms for metagenomic assembly has been elusive but also the assessment of genomic assemblers with metagenomic data has been overlooked systematically. Recently, Charuvaka et al have evaluated the assembly of short (36 bp) reads using simulated datasets similar to those used in our study. In their work, the authors assembled their data with the ABYSS assembler, but no functional characterization of the contigs was attempted [52]. This work seeks to throw some light on the difficult and sometimes unpredictable process of assembly metagenomic data sequenced with NGS technologies.

In the present study we used simulated reads obtained from already complete sequenced genomes (see Methods). This strategy allows us not only to know the origin of each read without being worried about contamination but also to use the genome annotations already available. In this way, we were able to compare the functional annotation of individual reads and contigs with that obtained from the completely assembled and annotated genomes. We have used a mixed strategy analyzing sets of sequences with and without the typical sequencing errors produced by different platforms. The error-free datasets may seem to provide an optimistic scenario although it has been reported that the majority of sequences coming from these platforms (more than 80% in the case of the 454 platform) are error-free with sequencing errors accumulating in a small proportion of the reads [39]. Moreover, quality filtering of the reads can increase substantially the proportion of perfect reads. By using the same datasets both with and without errors we are also able to analyze the impact of these errors in the assembly process and in their functional annotation. Interestingly, the Newbler assembler seems to deal particularly well with typical 454 errors and this is more pronounced when the assembler is fed in the native SFF format from Roche (data not shown).

It has also been proposed several strategies to alleviate typical problems in metagenomic assembly, like the pre-binning of the metagenomic reads based on sequence characteristics (for example frequency of n-mers) but to our knowledge this has not been rigorously tested to date. Rusch et al [53] have also proposed an “extreme assembly” method similar to a “greedy” algorithm where overlaps that allow the extension of the contigs are favored, but recruitment analysis to known genomes reveals the high amount of chimeric contigs obtained with this method. In viral metagenomes, it has been proposed the use of low-stringency assemblies to accommodate the genomic heterogeneity inherent in viral populations [54] reducing the number of viral types between three and five times.

Our results highlight some of the major problems of metagenome assembly. The degree of chimericity surpasses the 10% of the sampled “reads” in complex cases and because of the close taxonomical distance of the reads that forms these chimeric constructs, the pre-binning of the reads in OTUs prior to assembly is not expected to be an effective solution. Moreover, the assembly errors could prevent from a correct annotation of the contigs by lowering the sensitivity of homology searches. Annotation through profile databases like PFAM [55] or TIGRfam [56] may give better results and this possibility may be worth trying.

As expected, the factors that most influence the assembly quality are sample complexity, coverage and read length. A similar observation has been made before using shorter reads [52]. Sample complexity is inherent to the community under study and hence is not a variable in metagenomic experiments. The other two should be carefully revised when facing these kinds of projects. We have demonstrated that some of the problems in the annotation of high complex communities can be surpassed with an increase in the sequencing effort, while the use of longer reads will also help in their annotation.

In a previous study, Wommack et al have reported that a significant amount of short (100 bp–400 bp) sequences derived from longer Sanger reads (∼750 bp) missed distant homologies found with their longer counterparts [57]. Our results show a similar correlation between read length and functional annotation (Figure 3) and this is observed at the read and at the contig level with the longer contigs having better annotations than the shorter ones.

Results showed in Figure 2 also suggest to use only longer contigs for metagenome annotation since these have fewer errors when they are compared to their reference sequences. These long contigs, though, only account for a small proportion of the taxonomic and functional diversity of the sample. For this reason restricting the analysis to those long contigs could incur in annotation biases. We therefore advise against using only the longer contigs if a functional profile of the metagenome is the goal of the experiment.

At the present moment, the Illumina platform has a higher sequencing throughput than any pyrosequencing technology at a cheaper price with the hiseq2000 platform starting to work in genomic centers worldwide but apart from significant cases like the MetaHIT Consortium [34], the Illumina sequencer has not been extensively used in metagenomic projects. From our results it follows that at high coverage 110 bp dataset produces longer contigs with much less degree of chimericity than 400 bp datasets at lower coverage and these contigs contain less annotation errors. For instance, the 400 bp HC dataset produced 11.0 and 17.4 Mb of correctly annotated contigs assembled with Newbler and Celera respectively, while the 110 bp HChc dataset produced 171 Mbs and 275 Mbs (with SSAKE and VELVET respectively). Nevertheless, our results also suggest that the functional annotation of 400 bp datasets represents more accurately the functional content of the sampled genomes suggesting that coverage only may not substitute read length in this type of analysis.

## Methods

### Creation of simulated datasets

For each simulated dataset DNA fragments of the specified length were randomly selected from the complete set of 1012 completed genomes available at the NCBI site (as for February 2010). For each fragment different sampling information like the organism and chromosome of origin and its coordinates were recorded in a database for further traceability. Every fragment was also searched for identical sequences in all the genomes sampled in the same dataset. These identical alternative sites were also recorded as possible coordinates for each read. The simulated reads were reverse-complemented with a probability of 0.5.

### Simulation of sequencing errors

Typical next-generation sequencing errors were simulated for the metagenomes as follows. In the 454 error model, homopolymer length errors were introduced for the reads assuming that signals observed from a homopolymer of length *n* follow a Gaussian distribution with mean *n* and a standard deviation proportional to the square root of *n* with a coefficient of 0.15, while the light intensities for a negative flow follows a lognormal distribution with mean 0.23 and standard deviation of 0.15 [37], [38]. With this error model, we generated full SFF files used as input for the assembly process.

For the Illumina sequencing error, position dependent error rates have been reported before [40]. To simulate this kind of error, we approximate the average substitution rate using a model involving a fourth degree polynomial as described elsewhere [41]. We also included insertions and deletions with a probability of 0.0001% [40].

All datasets used in the present study (with and without sequencing errors) can be downloaded from the following URL:

http://metagenomics.uv.es/Supp/PONE2011_assemblers/

The program developed and used for the simulations (NGSfy) has been deposited in the public GitHub repository and can be obtained in the following url:

https://github.com/emepyc/NGSfy

### Assembly

Newbler assembler (version 2.3) was used with the following parameters “ml = 60 mi = 95 –ace”. The assembler was run several times with different values for “ml” (40 and 60) and mi (85, 90, 95 and 98) without impact in the conclusions described in this work. Celera assembler (version 6.1) was used with the following configuration: “utgErrorRate = 0.05, createACE = 1, merSize = 21, utgGenomeSize = 2000000, unitigger = BOG, overlapper = mer”. Most of them were suggested in the assembler documentation for metagenomic 454 data. Different values for utErrorRate (0.15, 0.1, 0.05 and 0.02) were also used without noticeable impact in the conclusions of the present work. Velvet (vesion 0.7) and SSAKE (version 3.4) were run with a word length of 23 nucleotides. Other values were also used (21 and 25) without noticeable impact in the final conclusions.

### Assembly evaluation

For each contig obtained, we traced each read back to determine all their possible positions in the reference genomes. We considered chimeric those contigs for which there were not possible to determine one single organism of origin. Those contigs were annotated as belonging to the organisms more represented in its reads solving ties by selecting one organism by random. For each chimeric contigs we also calculated the lower common ancestor (LCA) of its reads obtaining the level at which taxonomical integrity was preserved.

We also compared the resulting contigs with the reference genomes using the program BLAT [58] and calculated the percentage of identity of each contig with each matching reference.

### Metagenomic clustering

For the taxonomical trees we used the iTOL software [59] using the taxids of each sampled organism.

For the clustering based on whole-genome sequence similarity (Figure S4) we aligned all pairs of genomes used for sampling with the MAUVE software [60]. For each pair, we calculated the coverage of maximum unique matches (MUMs) without gaps in each genome of the pair and these values were used to construct a dissimilarity matrix. A hierarchical clustering was performed based on this matrix.

### Functional assignment

The COG corresponding to each simulated read was determined using the annotation of the genome from which the fragment was sampled. We used the chromosomal coordinates of each read to determine overlaps with annotated genes in the genome. The functional category of the most overlapping gene (with a minimum overlap of 40 bp with the read) was taken as the category of the read. We called this the “real annotation” of the read.

The functional category corresponding to each simulated read was also determined using BLASTx searches against the COG database [46] using an e-value cutoff of 10e^−3^. Each read was annotated with the functional category of the best hit. We called this the annotation at the read level.

After assembly, the functional category of each contig was determined using a similar strategy. Overlapping hits were merged together taking the best as the reference hit. We compared the contig coordinates of each read to assign them to a functional category. The functional category of the most overlapping hit (with a minimum overlap of 40 bp) was taken as the category of the read. We called this the annotation of the read at the contig level.

## Supporting Information

Figure S1 Taxonomical distribution of all organisms sampled in the simulated datasets (LC, MC and HC respectively). The labels indicate the taxid of each organism as represented in the NCBI database. Font colors for the labels represent the relative coverage of each genome.
(TIFF)

Figure S2 Taxonomic level of the lowest common ancestor of the chimeric contigs with platform specific errors. (A) 400 bp and (B) 110 bp datasets respectively. *N* stands for Newbler, *C* for Celera Assembler, *S* for SSAKE and *V* for Velvet.
(TIFF)

Figure S3 For the Newbler assembly of the MC dataset, heatmap representation of the percentage of reads of each pair of organisms sharing chimeric contigs. The color strip below the clusters indicates the relative coverage of each genome. The cladogram represents taxonomical relationship (based on the NCBI taxonomical classification) between the genomes sample for dataset MC. r1 and r2 identifies clusters of genomes that tend to form chimeric constructs during the assembly process and are also identified in Figure S4.
(TIFF)

Figure S4 Same figure as S3 but clustering the genomes based on whole-genome sequence alignments between each pair of genomes as explained in methods. Clusters r1 and r2 are the same clusters (i.e. formed by the same genomes) that were identified in Figure S4 although in this figure the resolution of r2 is much lower, probably because of the lower sensitivity of the clustering process.
(TIFF)

Figure S5 Same figure as Figure 3 but using simulated platform-specific sequencing errors.
(TIFF)

Figure S6 Same figure as Figure 4 but using simulated platform-specific sequencing errors.
(TIFF)

Table S1 Summary of the assembly statistics of the simulated datasets with platform-specific errors.
(DOC)

Dataset S1 Sampling information for the individual organisms used for the simulated datasets.
(DOC)
