# Supplementary figures and images for: Evaluating the Fidelity of De Novo Short Read Metagenomic Assembly Using Simulated Data
Source: PLoS One. 2011 May 23;6(5):e19984. doi: 10.1371/journal.pone.0019984 (PMC3100316; doi:10.1371/journal.pone.0019984)

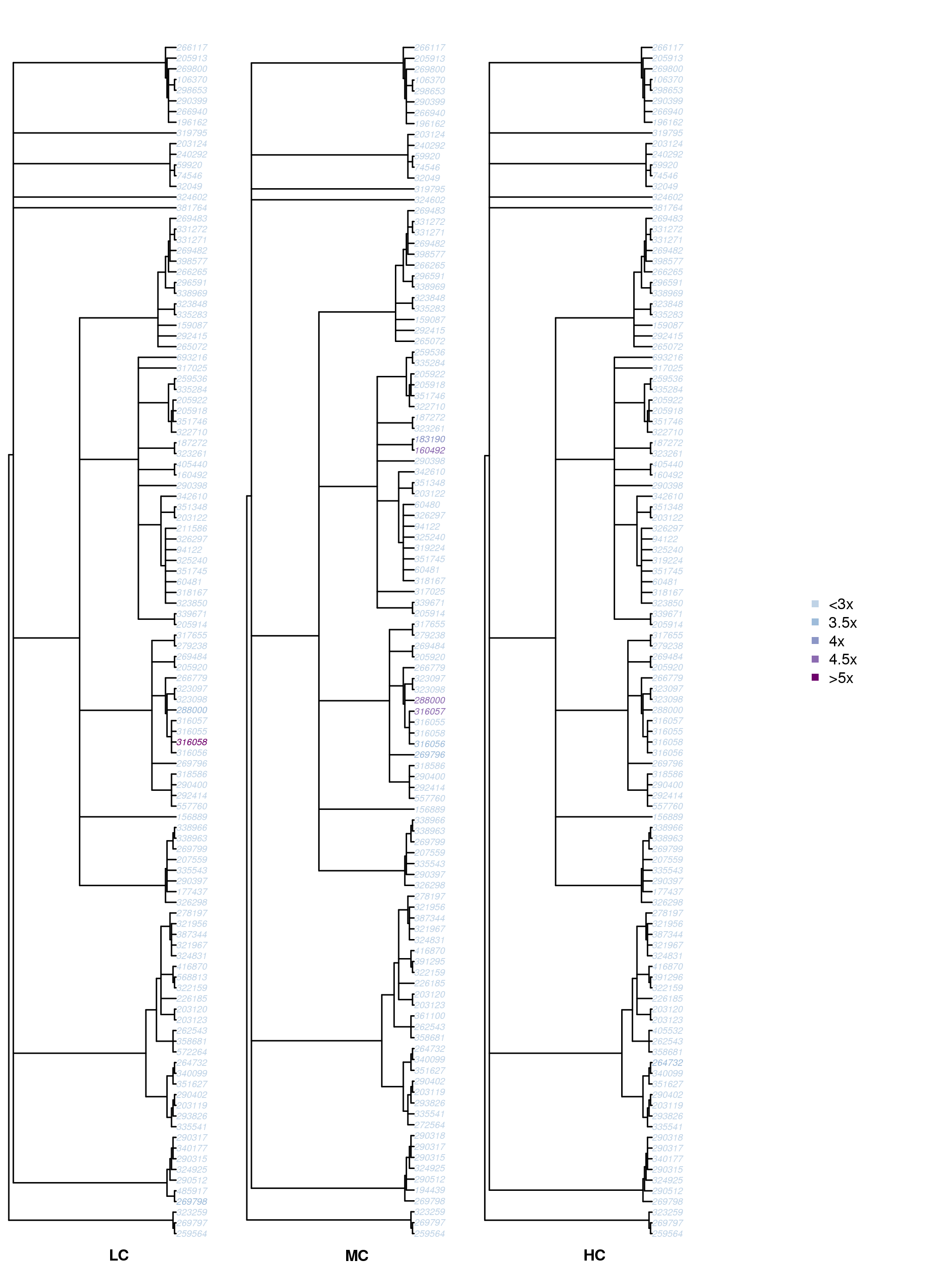

Supplement: Figure S1 — Taxonomical distribution of all organisms sampled in the simulated datasets (LC, MC and HC respectively). The labels indicate the taxid of each organism as represented in the NCBI database. Font colors for the labels represent the relative coverage of each genome. (TIFF) [file pone.0019984.s001.tif]

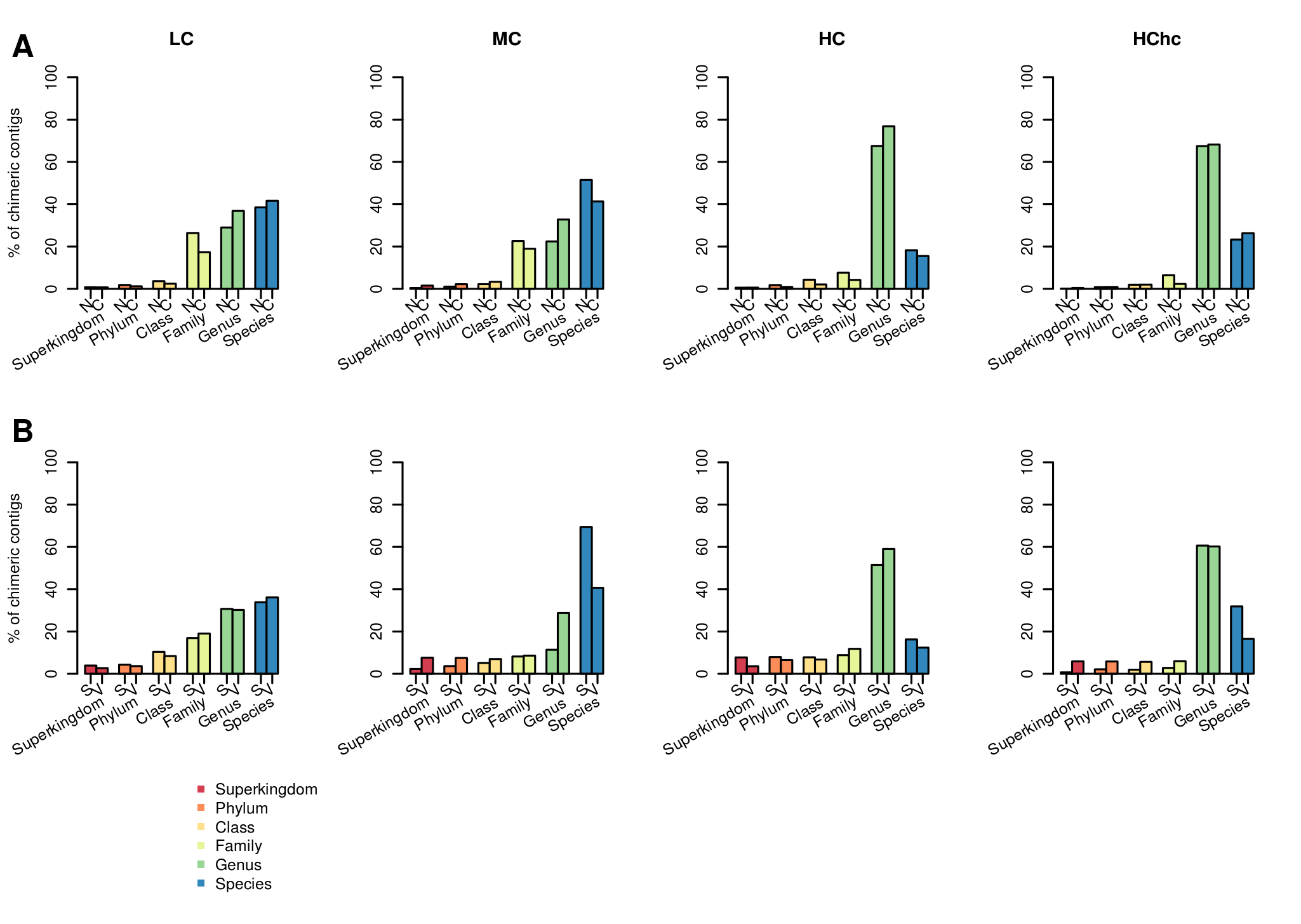

Supplement: Figure S2 — Taxonomic level of the lowest common ancestor of the chimeric contigs with platform specific errors. (A) 400 bp and (B) 110 bp datasets respectively. N stands for Newbler, C for Celera Assembler, S for SSAKE and V for Velvet. (TIFF) [file pone.0019984.s002.tif]

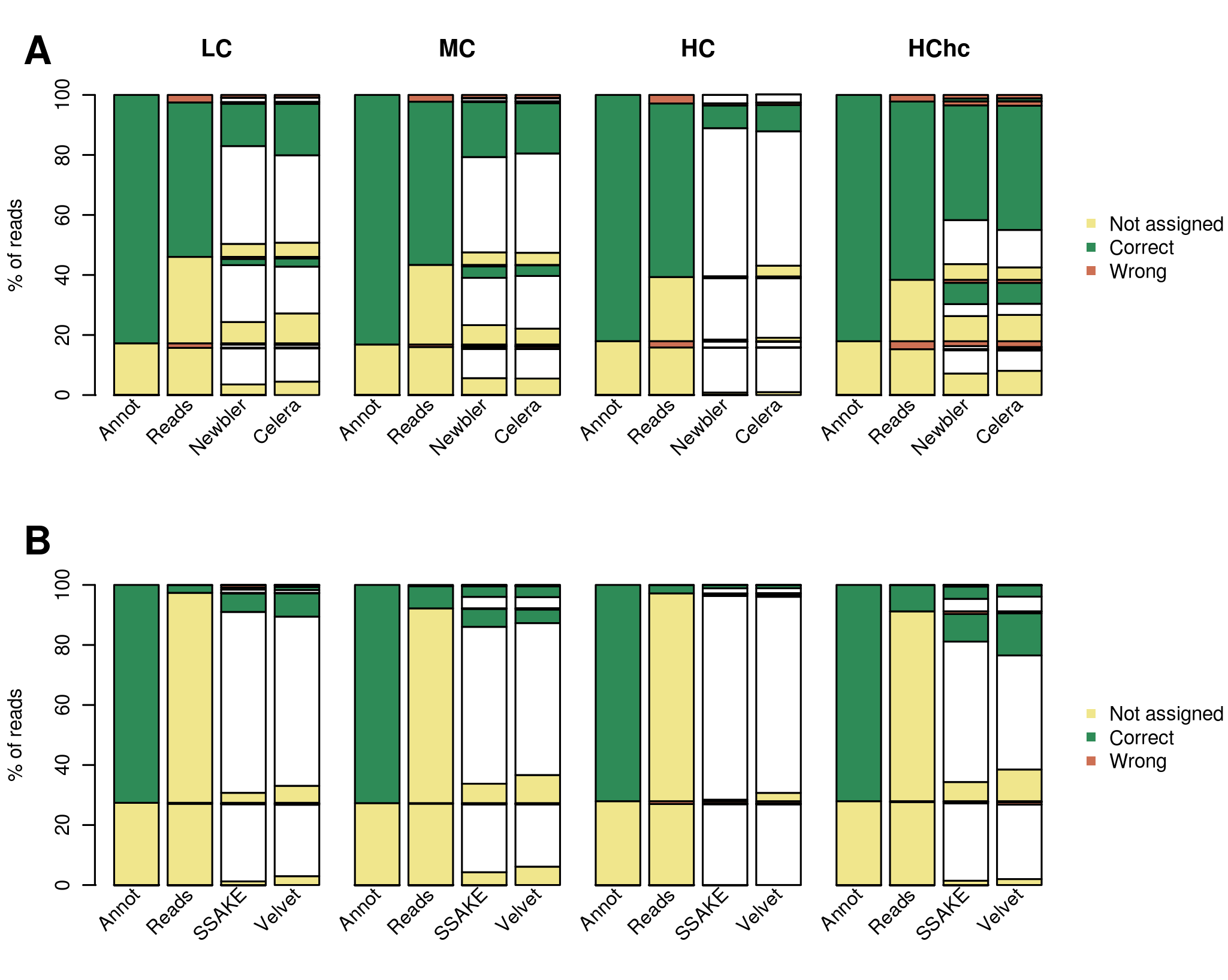

Supplement: Figure S5 — Same figure as Figure 3 but using simulated platform-specific sequencing errors. (TIFF) [file pone.0019984.s005.tif]

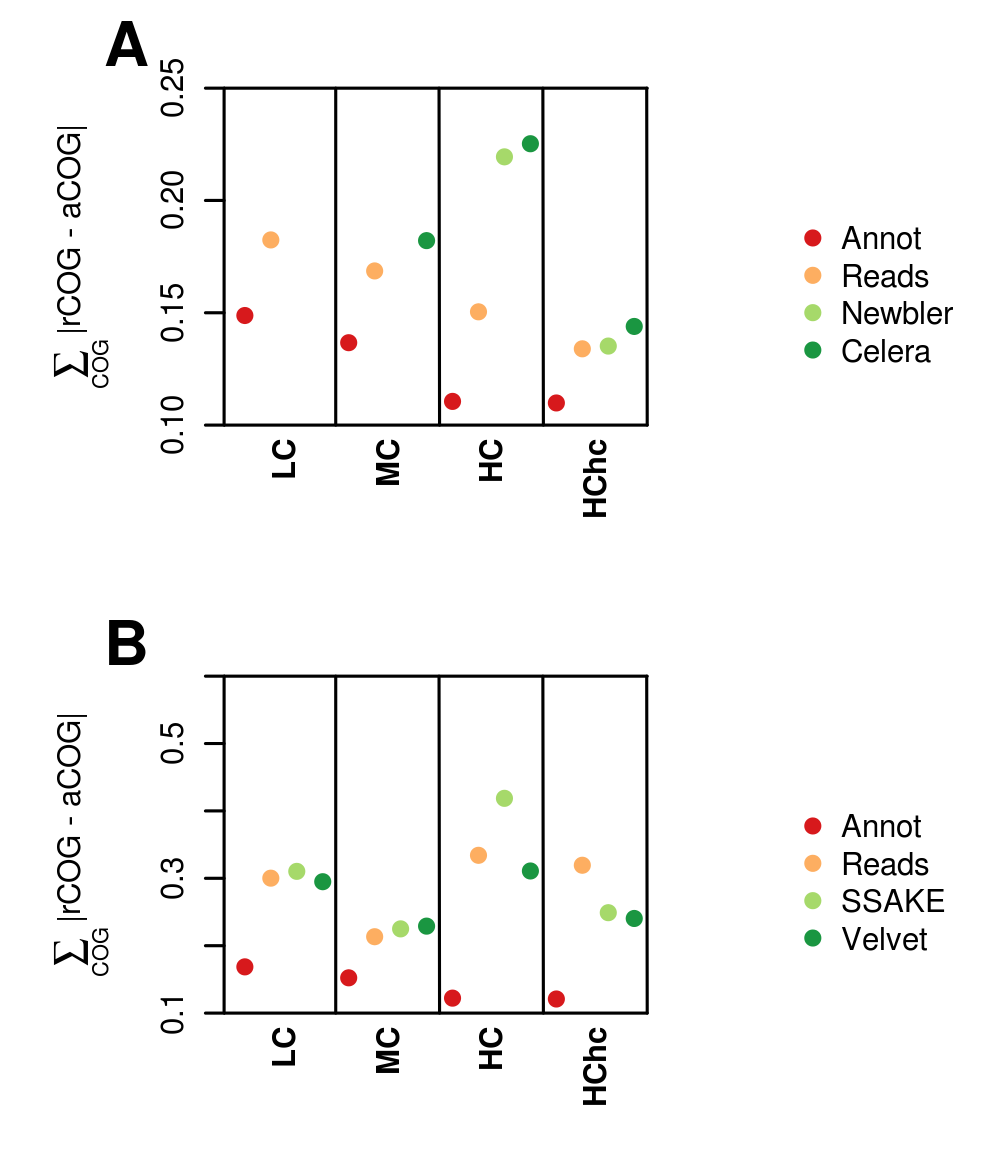

Supplement: Figure S6 — Same figure as Figure 4 but using simulated platform-specific sequencing errors. (TIFF) [file pone.0019984.s006.tif]
