# Supplementary material for: Evaluating the Fidelity of De Novo Short Read Metagenomic Assembly Using Simulated Data
Source: PLoS One. 2011 May 23;6(5):e19984. doi: 10.1371/journal.pone.0019984 (PMC3100316; doi:10.1371/journal.pone.0019984)
Supplement: Table S1 — Summary of the assembly statistics of the simulated datasets with platform-specific errors. (DOC) [file pone.0019984.s007.doc]

.

|  | **Assembler** | **LC** | | | **MC** | | | **HC** | | | **HChc** | | |
| --- | --- | --- | --- | --- | --- | --- | --- | --- | --- | --- | --- | --- | --- |
|  |  | **N50(bps)** | **Longest contig (bp)** | **% of reads in chimeras** | **N50(bps)** | **Longest contig (bp)** | **% of reads in chimeras** | **N50(bps)** | **Longest contig (bp)** | **% of reads in chimeras** | **N50(bps)** | **Longest contig (bp)** | **% of reads in chimeras** |
| **400bp** | **Newbler** | 3067 | 21052 | 4.02 | 1511 | 20919 | 10.09 | 457 | 2070 | 16.37 | 1213 | 31914 | 6.23 |
|  | **Celera** | 2661 | 15009 | 2.87 | 1056 | 11820 | 6.52 | 410 | 1112 | 11.29 | 1308 | 28919 | 6.61 |
| **110bp** | **SSAKE** | 177 | 1312 | 0.48 | 147 | 2323 | 4.33 | 108 | 994 | 8.12 | 144 | 3294 | 4.39 |
|  | **Velvet** | 141 | 1770 | 4.69 | 123 | 2010 | 9.88 | 100 | 802 | 8.88 | 132 | 3713 | 5.94 |

Only contigs longer than the read size were considered.
