# Supplementary material for: Evaluating the Fidelity of De Novo Short Read Metagenomic Assembly Using Simulated Data
Source: PLoS One. 2011 May 23;6(5):e19984. doi: 10.1371/journal.pone.0019984 (PMC3100316; doi:10.1371/journal.pone.0019984)
Supplement: Dataset S1 — Sampling information for the individual organisms used for the simulated datasets. (DOC) [file pone.0019984.s008.doc]

| **dataset** | **Organism** | **Chrom. size** | **Coverage** | **400bp reads** | **110bp reads** | **Chrom ID** | **Symbol** | **Taxonomy ID** |
| --- | --- | --- | --- | --- | --- | --- | --- | --- |
| SimLC | Rhodopseudomonas_palustris_HaA2 | 5331656 | 5.19 | 69178 | 251557 | NC_007778 | Rph | 316058 |
| SimLC | Bradyrhizobium_BTAi1 | 8264687 | 1.11 | 22935 | 83398 | NC_009485 | Bbt | 288000 |
| SimLC | Cytophaga_hutchinsonii_ATCC_33406 | 4433218 | 0.93 | 10307 | 37481 | NC_008255 | Cha | 269798 |
| SimLC | Xylella_fastidiosa | 2679306 | 0.44 | 2947 | 10717 | NC_002488 | Xfd | 160492 |
| SimLC | Moorella_thermoacetica_ATCC_39073 | 2628784 | 0.25 | 1643 | 5975 | NC_007644 | Mta | 264732 |
| SimLC | Xylella_fastidiosa_M12 | 2475130 | 0.2 | 1238 | 4500 | NC_010513 | Xfm | 405440 |
| SimLC | Ehrlichia_canis_Jake | 1315030 | 0.14 | 460 | 1674 | NC_007354 | Ecj | 269484 |
| SimLC | Rubrobacter_xylanophilus_DSM_9941 | 3225748 | 0.14 | 1129 | 4105 | NC_008148 | Rxd | 266117 |
| SimLC | Thiobacillus_denitrificans_ATCC_25259 | 2909809 | 0.14 | 1018 | 3703 | NC_007404 | Tda | 292415 |
| SimLC | Bacillus_cereus_03BB102 | 5269628 | 0.13 | 1713 | 6228 | NC_012472 | Bco | 572264 |
| SimLC | Burkholderia_383 | 3694126 | 0.13 | 1201 | 4366 | NC_007510 | Bur | 269483 |
| SimLC | Caldicellulosiruptor_saccharolyticus_DSM_8903 | 2970275 | 0.13 | 965 | 3510 | NC_009437 | Csa | 351627 |
| SimLC | Chloroflexus_aurantiacus_J_10_fl | 5258541 | 0.13 | 1709 | 6215 | NC_010175 | Caj | 324602 |
| SimLC | Clostridium_beijerinckii_NCIMB_8052 | 6000632 | 0.13 | 1950 | 7092 | NC_009617 | Cbn | 290402 |
| SimLC | Cronobacter_turicensis | 4384526 | 0.13 | 1425 | 5182 | NC_013282 | Ctu | 693216 |
| SimLC | Ehrlichia_chaffeensis_Arkansas | 1176248 | 0.13 | 382 | 1390 | NC_007799 | Eca | 205920 |
| SimLC | Prochlorococcus_marinus_NATL2A | 1842899 | 0.13 | 599 | 2178 | NC_007335 | Pmn | 59920 |
| SimLC | Psychrobacter_cryohalolentis_K5 | 3059876 | 0.13 | 994 | 3616 | NC_007969 | Pck | 335284 |
| SimLC | Rhodopseudomonas_palustris_BisB18 | 5513844 | 0.13 | 1792 | 6516 | NC_007925 | Rps | 316056 |
| SimLC | Shewanella_ANA-3 | 4972204 | 0.13 | 1616 | 5876 | NC_008577 | San | 94122 |
| SimLC | Shewanella_MR-7 | 4792610 | 0.13 | 1558 | 5664 | NC_008322 | Smr | 60481 |
| SimLC | Silicibacter_TM1040 | 3200938 | 0.13 | 1040 | 3783 | NC_008044 | Stm | 292414 |
| SimLC | Thermoanaerobacter_pseudethanolicus_ATCC_33223 | 2362816 | 0.13 | 768 | 2792 | NC_010321 | Tpa | 340099 |
| SimLC | Actinobacillus_succinogenes_130Z | 2319663 | 0.12 | 696 | 2531 | NC_009655 | Asg | 339671 |
| SimLC | Burkholderia_ambifaria_MC40_6 | 3443583 | 0.12 | 1033 | 3757 | NC_010551 | Bam | 398577 |
| SimLC | Chlorobium_limicola_DSM_245 | 2763181 | 0.12 | 829 | 3014 | NC_010803 | Cld | 290315 |
| SimLC | Deinococcus_geothermalis_DSM_11300 | 2467205 | 0.12 | 740 | 2691 | NC_008025 | Dgd | 319795 |
| SimLC | Jannaschia_CCS1 | 4317977 | 0.12 | 1295 | 4711 | NC_007802 | Jcc | 290400 |
| SimLC | Kineococcus_radiotolerans_SRS30216 | 4761183 | 0.12 | 1428 | 5194 | NC_009664 | Krs | 266940 |
| SimLC | Methylobacillus_flagellatus_KT | 2971517 | 0.12 | 891 | 3242 | NC_007947 | Mfk | 265072 |
| SimLC | Nitrobacter_winogradskyi_Nb-255 | 3402093 | 0.12 | 1021 | 3711 | NC_007406 | Nwn | 323098 |
| SimLC | Novosphingobium_aromaticivorans_DSM_12444 | 3561584 | 0.12 | 1068 | 3885 | NC_007794 | Nad | 279238 |
| SimLC | Pelodictyon_phaeoclathratiforme_BU_1 | 3018238 | 0.12 | 905 | 3293 | NC_011060 | Ppb | 324925 |
| SimLC | Polaromonas_JS666 | 5200264 | 0.12 | 1560 | 5673 | NC_007948 | Pjs | 296591 |
| SimLC | Pseudoalteromonas_atlantica_T6c | 5187005 | 0.12 | 1556 | 5659 | NC_008228 | Pat | 342610 |
| SimLC | Rhodopseudomonas_palustris_BisB5 | 4892717 | 0.12 | 1468 | 5338 | NC_007958 | Rpb | 316057 |
| SimLC | Sphingopyxis_alaskensis_RB2256 | 3345170 | 0.12 | 1004 | 3649 | NC_008048 | Spa | 317655 |
| SimLC | Thiomicrospira_denitrificans_ATCC_33889 | 2201561 | 0.12 | 660 | 2402 | NC_007575 | Tdt | 326298 |
| SimLC | Trichodesmium_erythraeum_IMS101 | 7750108 | 0.12 | 2325 | 8455 | NC_008312 | Tei | 203124 |
| SimLC | Alkalilimnicola_ehrlichei_MLHE-1 | 3275944 | 0.11 | 901 | 3276 | NC_008340 | Aem | 187272 |
| SimLC | Anabaena_variabilis_ATCC_29413 | 6365727 | 0.11 | 1751 | 6366 | NC_007413 | Ava | 240292 |
| SimLC | Anaeromyxobacter_dehalogenans_2CP-C | 5013479 | 0.11 | 1379 | 5013 | NC_007760 | Adc | 290397 |
| SimLC | Arthrobacter_FB24 | 4698945 | 0.11 | 1292 | 4699 | NC_008541 | Afb | 290399 |
| SimLC | Azotobacter_vinelandii_DJ | 5365318 | 0.11 | 1475 | 5365 | NC_012560 | Avd | 322710 |
| SimLC | Burkholderia_cenocepacia_AU_1054 | 3294563 | 0.11 | 906 | 3295 | NC_008060 | Bca | 331271 |
| SimLC | Burkholderia_cenocepacia_HI2424 | 3483902 | 0.11 | 958 | 3484 | NC_008542 | Bch | 331272 |
| SimLC | Burkholderia_vietnamiensis_G4 | 3652814 | 0.11 | 1005 | 3653 | NC_009256 | Bvg | 269482 |
| SimLC | Clostridium_thermocellum_ATCC_27405 | 3843301 | 0.11 | 1057 | 3843 | NC_009012 | Cta | 203119 |
| SimLC | Desulfobacterium_autotrophicum_HRM2 | 5589073 | 0.11 | 1537 | 5589 | NC_012108 | Dah | 177437 |
| SimLC | Desulfovibrio_desulfuricans_G20 | 3730232 | 0.11 | 1026 | 3730 | NC_007519 | Ddg | 207559 |
| SimLC | Exiguobacterium_sibiricum_255_15 | 3034136 | 0.11 | 834 | 3034 | NC_010556 | Esi | 262543 |
| SimLC | Frankia_CcI3 | 5433628 | 0.11 | 1494 | 5434 | NC_007777 | Fcc | 106370 |
| SimLC | Frankia_EAN1pec | 8982042 | 0.11 | 2470 | 8982 | NC_009921 | Fea | 298653 |
| SimLC | Geobacter_metallireducens_GS-15 | 3997420 | 0.11 | 1099 | 3997 | NC_007517 | Gmg | 269799 |
| SimLC | Lactobacillus_casei_ATCC_334 | 2895264 | 0.11 | 796 | 2895 | NC_008526 | Lca | 321967 |
| SimLC | Lactobacillus_gasseri_ATCC_33323 | 1894360 | 0.11 | 521 | 1894 | NC_008530 | Lga | 324831 |
| SimLC | Marinobacter_aquaeolei_VT8 | 4326849 | 0.11 | 1190 | 4327 | NC_008740 | Mav | 351348 |
| SimLC | Methanospirillum_hungatei_JF-1 | 3544738 | 0.11 | 975 | 3545 | NC_007796 | Mhj | 323259 |
| SimLC | Nitrobacter_hamburgensis_X14 | 4406967 | 0.11 | 1212 | 4407 | NC_007964 | Nhx | 323097 |
| SimLC | Nitrosococcus_oceani_ATCC_19707 | 3481691 | 0.11 | 957 | 3482 | NC_007484 | Noa | 323261 |
| SimLC | Nitrosomonas_eutropha_C71 | 2661057 | 0.11 | 732 | 2661 | NC_008344 | Nec | 335283 |
| SimLC | Nitrosospira_multiformis_ATCC_25196 | 3184243 | 0.11 | 876 | 3184 | NC_007614 | Nma | 323848 |
| SimLC | Nocardioides_JS614 | 4985871 | 0.11 | 1371 | 4986 | NC_008699 | Njs | 196162 |
| SimLC | Pelobacter_carbinolicus | 3665893 | 0.11 | 1008 | 3666 | NC_007498 | Pca | 338963 |
| SimLC | Pelobacter_propionicus_DSM_2379 | 4008000 | 0.11 | 1102 | 4008 | NC_008609 | Ppd | 338966 |
| SimLC | Pseudomonas_putida_F1 | 5959964 | 0.11 | 1639 | 5960 | NC_009512 | Ppf | 351746 |
| SimLC | Pseudomonas_syringae_pv_B728a | 6093698 | 0.11 | 1676 | 6094 | NC_007005 | Psp | 205918 |
| SimLC | Rhodoferax_ferrireducens_T118 | 4712337 | 0.11 | 1296 | 4712 | NC_007908 | Rft | 338969 |
| SimLC | Rhodopseudomonas_palustris_BisA53 | 5505494 | 0.11 | 1514 | 5505 | NC_008435 | Rpi | 316055 |
| SimLC | Rhodospirillum_rubrum_ATCC_11170 | 4352825 | 0.11 | 1197 | 4353 | NC_007643 | Rra | 269796 |
| SimLC | Shewanella_amazonensis_SB2B | 4306142 | 0.11 | 1184 | 4306 | NC_008700 | Sas | 326297 |
| SimLC | Shewanella_baltica_OS155 | 5127376 | 0.11 | 1410 | 5127 | NC_009052 | Sbo | 325240 |
| SimLC | Shewanella_frigidimarina_NCIMB_400 | 4845257 | 0.11 | 1332 | 4845 | NC_008345 | Sgn | 318167 |
| SimLC | Shewanella_oneidensis | 4969803 | 0.11 | 1367 | 4970 | NC_004347 | Son | 211586 |
| SimLC | Shewanella_loihica_PV-4 | 4602594 | 0.11 | 1266 | 4603 | NC_009092 | Slp | 323850 |
| SimLC | Streptococcus_suis_SC84 | 2095898 | 0.11 | 576 | 2096 | NC_012924 | Sss | 568813 |
| SimLC | Syntrophobacter_fumaroxidans_MPOB | 4990251 | 0.11 | 1372 | 4990 | NC_008554 | Sfm | 335543 |
| SimLC | Alkaliphilus_metalliredigens_QYMF | 4929566 | 0.1 | 1232 | 4481 | NC_009633 | Amq | 293826 |
| SimLC | Bifidobacterium_longum_DJO10A | 2375792 | 0.1 | 594 | 2160 | NC_010816 | Bld | 205913 |
| SimLC | Brevibacillus_brevis_NBRC_100599 | 6296436 | 0.1 | 1574 | 5724 | NC_012491 | Bbn | 358681 |
| SimLC | Chlorobium_phaeobacteroides_DSM_266 | 3133902 | 0.1 | 783 | 2849 | NC_008639 | Cpd | 290317 |
| SimLC | Dechloromonas_aromatica_RCB | 4501104 | 0.1 | 1125 | 4092 | NC_007298 | Dar | 159087 |
| SimLC | Fervidobacterium_nodosum_Rt17-B1 | 1948941 | 0.1 | 487 | 1772 | NC_009718 | Fnr | 381764 |
| SimLC | Haemophilus_somnus_129PT | 2007700 | 0.1 | 502 | 1825 | NC_008309 | Hso | 205914 |
| SimLC | Lactococcus_lactis_cremoris_MG1363 | 2529478 | 0.1 | 632 | 2300 | NC_009004 | Llc | 416870 |
| SimLC | Leuconostoc_mesenteroides_ATCC_8293 | 2038396 | 0.1 | 510 | 1853 | NC_008531 | Lma | 203120 |
| SimLC | Magnetococcus_MC-1 | 4719581 | 0.1 | 1180 | 4291 | NC_008576 | Mmc | 156889 |
| SimLC | Paracoccus_denitrificans_PD1222 | 2852282 | 0.1 | 713 | 2593 | NC_008686 | Pdp | 318586 |
| SimLC | Pediococcus_pentosaceus_ATCC_25745 | 1832387 | 0.1 | 458 | 1666 | NC_008525 | Ppa | 278197 |
| SimLC | Pedobacter_heparinus_DSM_2366 | 5167383 | 0.1 | 1292 | 4698 | NC_013061 | Phd | 485917 |
| SimLC | Prochlorococcus_marinus_MIT_9312 | 1709204 | 0.1 | 427 | 1554 | NC_007577 | Pmm | 74546 |
| SimLC | Prosthecochloris_aestuarii_DSM_271 | 2512923 | 0.1 | 628 | 2284 | NC_011059 | Pad | 290512 |
| SimLC | Saccharophagus_degradans_2-40 | 5057531 | 0.1 | 1264 | 4598 | NC_007912 | Sde | 203122 |
| SimLC | Shewanella_W3-18-1 | 4708380 | 0.1 | 1177 | 4280 | NC_008750 | Shw | 351745 |
| SimLC | Syntrophomonas_wolfei_Goettingen | 2936195 | 0.1 | 734 | 2669 | NC_008346 | Swg | 335541 |
| SimLC | Thiomicrospira_crunogena_XCL-2 | 2427734 | 0.1 | 607 | 2207 | NC_007520 | Tcx | 317025 |
| SimLC | Burkholderia_xenovorans_LB400 | 4895836 | 0.09 | 1102 | 4006 | NC_007951 | Bxl | 266265 |
| SimLC | Chlorobium_chlorochromatii_CaD3 | 2572079 | 0.09 | 579 | 2104 | NC_007514 | Ccc | 340177 |
| SimLC | Chromohalobacter_salexigens_DSM_3043 | 3696649 | 0.09 | 832 | 3025 | NC_007963 | Csd | 290398 |
| SimLC | Enterococcus_faecalis_V583 | 3218031 | 0.09 | 724 | 2633 | NC_004668 | Efv | 226185 |
| SimLC | Lactobacillus_delbrueckii_bulgaricus_ATCC_BAA-365 | 1856951 | 0.09 | 418 | 1519 | NC_008529 | Ldb | 321956 |
| SimLC | Mesorhizobium_BNC1 | 4412446 | 0.09 | 993 | 3610 | NC_008254 | Mbn | 266779 |
| SimLC | Methanococcoides_burtonii_DSM_6242 | 2575032 | 0.09 | 579 | 2107 | NC_007955 | Mbd | 259564 |
| SimLC | Methanosarcina_barkeri_fusaro | 4837408 | 0.09 | 1088 | 3958 | NC_007355 | Mbf | 269797 |
| SimLC | Pseudomonas_fluorescens_Pf0_1 | 6438405 | 0.09 | 1449 | 5268 | NC_007492 | Pfp | 205922 |
| SimLC | Psychrobacter_arcticum_273-4 | 2650701 | 0.09 | 596 | 2169 | NC_007204 | Par | 259536 |
| SimLC | Synechococcus_PCC_7002 | 3008047 | 0.09 | 677 | 2461 | NC_010475 | Syp | 32049 |
| SimLC | Thermobifida_fusca_YX | 3642249 | 0.09 | 820 | 2980 | NC_007333 | Tfy | 269800 |
| SimLC | Oenococcus_oeni_PSU-1 | 1780517 | 0.08 | 356 | 1295 | NC_008528 | Oop | 203123 |
| SimLC | Rhodobacter_sphaeroides_KD131 | 3152792 | 0.08 | 631 | 2293 | NC_011963 | Rsk | 557760 |
| SimLC | Lactobacillus_brevis_ATCC_367 | 2291220 | 0.07 | 401 | 1458 | NC_008497 | Lba | 387344 |
| SimLC | Streptococcus_thermophilus_LMD-9 | 1856368 | 0.07 | 325 | 1181 | NC_008532 | Stl | 322159 |
| SimMC | Xylella_fastidiosa | 2679306 | 3.48 | 23310 | 84763 | NC_002488 | Xfd | 160492 |
| SimMC | Rhodopseudomonas_palustris_BisB5 | 4892717 | 3.47 | 42444 | 154343 | NC_007958 | Rpb | 316057 |
| SimMC | Bradyrhizobium_BTAi1 | 8264687 | 2.77 | 57233 | 208120 | NC_009485 | Bbt | 288000 |
| SimMC | Xylella_fastidiosa_Temecula1 | 2519802 | 1.68 | 10583 | 38484 | NC_004556 | Xft | 183190 |
| SimMC | Rhodopseudomonas_palustris_BisB18 | 5513844 | 1.13 | 15577 | 56642 | NC_007925 | Rps | 316056 |
| SimMC | Rhodospirillum_rubrum_ATCC_11170 | 4352825 | 1.02 | 11100 | 40363 | NC_007643 | Rra | 269796 |
| SimMC | Moorella_thermoacetica_ATCC_39073 | 2628784 | 0.28 | 1840 | 6691 | NC_007644 | Mta | 264732 |
| SimMC | Rubrobacter_xylanophilus_DSM_9941 | 3225748 | 0.15 | 1210 | 4399 | NC_008148 | Rxd | 266117 |
| SimMC | Thiobacillus_denitrificans_ATCC_25259 | 2909809 | 0.15 | 1091 | 3968 | NC_007404 | Tda | 292415 |
| SimMC | Actinobacillus_succinogenes_130Z | 2319663 | 0.14 | 812 | 2952 | NC_009655 | Asg | 339671 |
| SimMC | Ehrlichia_canis_Jake | 1315030 | 0.14 | 460 | 1674 | NC_007354 | Ecj | 269484 |
| SimMC | Ehrlichia_chaffeensis_Arkansas | 1176248 | 0.14 | 412 | 1497 | NC_007799 | Eca | 205920 |
| SimMC | Chlorobium_tepidum_TLS | 2154946 | 0.13 | 700 | 2547 | NC_002932 | Ctt | 194439 |
| SimMC | Polaromonas_JS666 | 5200264 | 0.13 | 1690 | 6146 | NC_007948 | Pjs | 296591 |
| SimMC | Pseudoalteromonas_atlantica_T6c | 5187005 | 0.13 | 1686 | 6130 | NC_008228 | Pat | 342610 |
| SimMC | Silicibacter_TM1040 | 3200938 | 0.13 | 1040 | 3783 | NC_008044 | Stm | 292414 |
| SimMC | Thiomicrospira_denitrificans_ATCC_33889 | 2201561 | 0.13 | 716 | 2602 | NC_007575 | Tdt | 326298 |
| SimMC | Burkholderia_ambifaria_MC40_6 | 3443583 | 0.12 | 1033 | 3757 | NC_010551 | Bam | 398577 |
| SimMC | Burkholderia_383 | 3694126 | 0.12 | 1108 | 4030 | NC_007510 | Bur | 269483 |
| SimMC | Chlorobium_limicola_DSM_245 | 2763181 | 0.12 | 829 | 3014 | NC_010803 | Cld | 290315 |
| SimMC | Chloroflexus_aurantiacus_J_10_fl | 5258541 | 0.12 | 1578 | 5737 | NC_010175 | Caj | 324602 |
| SimMC | Clostridium_beijerinckii_NCIMB_8052 | 6000632 | 0.12 | 1800 | 6546 | NC_009617 | Cbn | 290402 |
| SimMC | Clostridium_thermocellum_ATCC_27405 | 3843301 | 0.12 | 1153 | 4193 | NC_009012 | Cta | 203119 |
| SimMC | Deinococcus_geothermalis_DSM_11300 | 2467205 | 0.12 | 740 | 2691 | NC_008025 | Dgd | 319795 |
| SimMC | Jannaschia_CCS1 | 4317977 | 0.12 | 1295 | 4711 | NC_007802 | Jcc | 290400 |
| SimMC | Kineococcus_radiotolerans_SRS30216 | 4761183 | 0.12 | 1428 | 5194 | NC_009664 | Krs | 266940 |
| SimMC | Lactobacillus_gasseri_ATCC_33323 | 1894360 | 0.12 | 568 | 2067 | NC_008530 | Lga | 324831 |
| SimMC | Lactococcus_lactis_cremoris_MG1363 | 2529478 | 0.12 | 759 | 2759 | NC_009004 | Llc | 416870 |
| SimMC | Marinobacter_aquaeolei_VT8 | 4326849 | 0.12 | 1298 | 4720 | NC_008740 | Mav | 351348 |
| SimMC | Methanospirillum_hungatei_JF-1 | 3544738 | 0.12 | 1063 | 3867 | NC_007796 | Mhj | 323259 |
| SimMC | Nitrosospira_multiformis_ATCC_25196 | 3184243 | 0.12 | 955 | 3474 | NC_007614 | Nma | 323848 |
| SimMC | Nocardioides_JS614 | 4985871 | 0.12 | 1496 | 5439 | NC_008699 | Njs | 196162 |
| SimMC | Novosphingobium_aromaticivorans_DSM_12444 | 3561584 | 0.12 | 1068 | 3885 | NC_007794 | Nad | 279238 |
| SimMC | Pelobacter_propionicus_DSM_2379 | 4008000 | 0.12 | 1202 | 4372 | NC_008609 | Ppd | 338966 |
| SimMC | Pelodictyon_phaeoclathratiforme_BU_1 | 3018238 | 0.12 | 905 | 3293 | NC_011060 | Ppb | 324925 |
| SimMC | Prochlorococcus_marinus_MIT_9312 | 1709204 | 0.12 | 513 | 1865 | NC_007577 | Pmm | 74546 |
| SimMC | Pseudomonas_putida_F1 | 5959964 | 0.12 | 1788 | 6502 | NC_009512 | Ppf | 351746 |
| SimMC | Rhodopseudomonas_palustris_BisA53/ | 5505494 | 0.12 | 1652 | 6006 | NC_008435 | Rpa | 316055 |
| SimMC | Rhodopseudomonas_palustris_HaA2 | 5331656 | 0.12 | 1599 | 5816 | NC_007778 | Rph | 316058 |
| SimMC | Shewanella_baltica_OS155 | 5127376 | 0.12 | 1538 | 5594 | NC_009052 | Sbo | 325240 |
| SimMC | Streptococcus_suis_05ZYH33 | 2096309 | 0.12 | 629 | 2287 | NC_009442 | Sso | 391295 |
| SimMC | Syntrophomonas_wolfei_Goettingen | 2936195 | 0.12 | 881 | 3203 | NC_008346 | Swg | 335541 |
| SimMC | Alkalilimnicola_ehrlichei_MLHE-1 | 3275944 | 0.11 | 901 | 3276 | NC_008340 | Aem | 187272 |
| SimMC | Anabaena_variabilis_ATCC_29413 | 6365727 | 0.11 | 1751 | 6366 | NC_007413 | Ava | 240292 |
| SimMC | Anaeromyxobacter_dehalogenans_2CP-C | 5013479 | 0.11 | 1379 | 5013 | NC_007760 | Adc | 290397 |
| SimMC | Arthrobacter_FB24 | 4698945 | 0.11 | 1292 | 4699 | NC_008541 | Afb | 290399 |
| SimMC | Azotobacter_vinelandii_DJ | 5365318 | 0.11 | 1475 | 5365 | NC_012560 | Avd | 322710 |
| SimMC | Bacillus_cereus_Q1 | 5214195 | 0.11 | 1434 | 5214 | NC_011969 | Bcq | 361100 |
| SimMC | Burkholderia_cenocepacia_AU_1054 | 3294563 | 0.11 | 906 | 3295 | NC_008060 | Bca | 331271 |
| SimMC | Burkholderia_cenocepacia_HI2424 | 3483902 | 0.11 | 958 | 3484 | NC_008542 | Bch | 331272 |
| SimMC | Burkholderia_vietnamiensis_G4 | 3652814 | 0.11 | 1005 | 3653 | NC_009256 | Bvg | 269482 |
| SimMC | Caldicellulosiruptor_saccharolyticus_DSM_8903 | 2970275 | 0.11 | 817 | 2970 | NC_009437 | Csa | 351627 |
| SimMC | Chlorobium_phaeobacteroides_DSM_266 | 3133902 | 0.11 | 862 | 3134 | NC_008639 | Cpd | 290317 |
| SimMC | Exiguobacterium_sibiricum_255_15 | 3034136 | 0.11 | 834 | 3034 | NC_010556 | Esi | 262543 |
| SimMC | Frankia_CcI3 | 5433628 | 0.11 | 1494 | 5434 | NC_007777 | Fcc | 106370 |
| SimMC | Frankia_EAN1pec | 8982042 | 0.11 | 2470 | 8982 | NC_009921 | Fea | 298653 |
| SimMC | Geobacter_metallireducens_GS-15 | 3997420 | 0.11 | 1099 | 3997 | NC_007517 | Gmg | 269799 |
| SimMC | Haemophilus_somnus_129PT | 2007700 | 0.11 | 552 | 2008 | NC_008309 | Hso | 205914 |
| SimMC | Lactobacillus_casei_ATCC_334 | 2895264 | 0.11 | 796 | 2895 | NC_008526 | Lca | 321967 |
| SimMC | Methanococcoides_burtonii_DSM_6242 | 2575032 | 0.11 | 708 | 2575 | NC_007955 | Mbd | 259564 |
| SimMC | Nitrosococcus_oceani_ATCC_19707 | 3481691 | 0.11 | 957 | 3482 | NC_007484 | Noa | 323261 |
| SimMC | Nitrosomonas_eutropha_C71 | 2661057 | 0.11 | 732 | 2661 | NC_008344 | Nec | 335283 |
| SimMC | Paracoccus_denitrificans_PD1222 | 2852282 | 0.11 | 784 | 2852 | NC_008686 | Pdp | 318586 |
| SimMC | Pelobacter_carbinolicus | 3665893 | 0.11 | 1008 | 3666 | NC_007498 | Pca | 338963 |
| SimMC | Psychrobacter_cryohalolentis_K5 | 3059876 | 0.11 | 841 | 3060 | NC_007969 | Pck | 335284 |
| SimMC | Rhodoferax_ferrireducens_T118 | 4712337 | 0.11 | 1296 | 4712 | NC_007908 | Rft | 338969 |
| SimMC | Saccharophagus_degradans_2-40 | 5057531 | 0.11 | 1391 | 5058 | NC_007912 | Sde | 203122 |
| SimMC | Shewanella_frigidimarina_NCIMB_400 | 4845257 | 0.11 | 1332 | 4845 | NC_008345 | Sgn | 318167 |
| SimMC | Shewanella_putrefaciens_CN-32 | 4659220 | 0.11 | 1281 | 4659 | NC_009438 | Spc | 319224 |
| SimMC | Shewanella_ANA-3 | 4972204 | 0.11 | 1367 | 4972 | NC_008577 | San | 94122 |
| SimMC | Shewanella_MR-7 | 4792610 | 0.11 | 1318 | 4793 | NC_008322 | Smr | 60481 |
| SimMC | Shewanella_MR-4 | 4706287 | 0.11 | 1294 | 4706 | NC_008321 | Shm | 60480 |
| SimMC | Shewanella_W3-18-1 | 4708380 | 0.11 | 1295 | 4708 | NC_008750 | Shw | 351745 |
| SimMC | Sphingopyxis_alaskensis_RB2256 | 3345170 | 0.11 | 920 | 3345 | NC_008048 | Spa | 317655 |
| SimMC | Thiomicrospira_crunogena_XCL-2 | 2427734 | 0.11 | 668 | 2428 | NC_007520 | Tcx | 317025 |
| SimMC | Trichodesmium_erythraeum_IMS101 | 7750108 | 0.11 | 2131 | 7750 | NC_008312 | Tei | 203124 |
| SimMC | Alkaliphilus_metalliredigens_QYMF | 4929566 | 0.1 | 1232 | 4481 | NC_009633 | Amq | 293826 |
| SimMC | Chromohalobacter_salexigens_DSM_3043 | 3696649 | 0.1 | 924 | 3361 | NC_007963 | Csd | 290398 |
| SimMC | Cytophaga_hutchinsonii_ATCC_33406 | 4433218 | 0.1 | 1108 | 4030 | NC_008255 | Cha | 269798 |
| SimMC | Dechloromonas_aromatica_RCB | 4501104 | 0.1 | 1125 | 4092 | NC_007298 | Dar | 159087 |
| SimMC | Desulfitobacterium_hafniense_DCB_2 | 5279134 | 0.1 | 1320 | 4799 | NC_011830 | Deh | 272564 |
| SimMC | Desulfovibrio_desulfuricans_G20 | 3730232 | 0.1 | 933 | 3391 | NC_007519 | Ddg | 207559 |
| SimMC | Lactobacillus_brevis_ATCC_367 | 2291220 | 0.1 | 573 | 2083 | NC_008497 | Lba | 387344 |
| SimMC | Leuconostoc_mesenteroides_ATCC_8293 | 2038396 | 0.1 | 510 | 1853 | NC_008531 | Lma | 203120 |
| SimMC | Magnetococcus_MC-1 | 4719581 | 0.1 | 1180 | 4291 | NC_008576 | Mmc | 156889 |
| SimMC | Mesorhizobium_BNC1 | 4412446 | 0.1 | 1103 | 4011 | NC_008254 | Mbn | 266779 |
| SimMC | Methanosarcina_barkeri_fusaro | 4837408 | 0.1 | 1209 | 4398 | NC_007355 | Mbf | 269797 |
| SimMC | Methylobacillus_flagellatus_KT | 2971517 | 0.1 | 743 | 2701 | NC_007947 | Mfk | 265072 |
| SimMC | Nitrobacter_hamburgensis_X14 | 4406967 | 0.1 | 1102 | 4006 | NC_007964 | Nhx | 323097 |
| SimMC | Nitrobacter_winogradskyi_Nb-255 | 3402093 | 0.1 | 851 | 3093 | NC_007406 | Nwn | 323098 |
| SimMC | Oenococcus_oeni_PSU-1 | 1780517 | 0.1 | 445 | 1619 | NC_008528 | Oop | 203123 |
| SimMC | Prosthecochloris_aestuarii_DSM_271 | 2512923 | 0.1 | 628 | 2284 | NC_011059 | Pad | 290512 |
| SimMC | Prosthecochloris_vibrioformis_DSM_265 | 1966858 | 0.1 | 492 | 1788 | NC_009337 | Pvd | 290318 |
| SimMC | Pseudomonas_syringae_pv_B728a | 6093698 | 0.1 | 1523 | 5540 | NC_007005 | Psp | 205918 |
| SimMC | Shewanella_amazonensis_SB2B | 4306142 | 0.1 | 1077 | 3915 | NC_008700 | Sas | 326297 |
| SimMC | Synechococcus_PCC_7002 | 3008047 | 0.1 | 752 | 2735 | NC_010475 | Syp | 32049 |
| SimMC | Syntrophobacter_fumaroxidans_MPOB | 4990251 | 0.1 | 1248 | 4537 | NC_008554 | Sfm | 335543 |
| SimMC | Thermoanaerobacter_pseudethanolicus_ATCC_33223 | 2362816 | 0.1 | 591 | 2148 | NC_010321 | Tpa | 340099 |
| SimMC | Brevibacillus_brevis_NBRC_100599 | 6296436 | 0.09 | 1417 | 5152 | NC_012491 | Bbn | 358681 |
| SimMC | Burkholderia_xenovorans_LB400 | 4895836 | 0.09 | 1102 | 4006 | NC_007951 | Bxl | 266265 |
| SimMC | Enterococcus_faecalis_V583 | 3218031 | 0.09 | 724 | 2633 | NC_004668 | Efv | 226185 |
| SimMC | Lactobacillus_delbrueckii_bulgaricus_ATCC_BAA-365 | 1856951 | 0.09 | 418 | 1519 | NC_008529 | Ldb | 321956 |
| SimMC | Prochlorococcus_marinus_NATL2A | 1842899 | 0.09 | 415 | 1508 | NC_007335 | Pmn | 59920 |
| SimMC | Pseudomonas_fluorescens_Pf0_1 | 6438405 | 0.09 | 1449 | 5268 | NC_007492 | Pfp | 205922 |
| SimMC | Psychrobacter_arcticum_273-4 | 2650701 | 0.09 | 596 | 2169 | NC_007204 | Par | 259536 |
| SimMC | Thermobifida_fusca_YX | 3642249 | 0.09 | 820 | 2980 | NC_007333 | Tfy | 269800 |
| SimMC | Bifidobacterium_longum_DJO10A | 2375792 | 0.08 | 475 | 1728 | NC_010816 | Bld | 205913 |
| SimMC | Pediococcus_pentosaceus_ATCC_25745 | 1832387 | 0.08 | 366 | 1333 | NC_008525 | Ppa | 278197 |
| SimMC | Rhodobacter_sphaeroides_KD131 | 3152792 | 0.08 | 631 | 2293 | NC_011963 | Rsk | 557760 |
| SimMC | Streptococcus_thermophilus_LMD-9 | 1856368 | 0.08 | 371 | 1350 | NC_008532 | Stl | 322159 |
| SimHC | Moorella_thermoacetica_ATCC_39073 | 2628784 | 0.53 | 3483 | 12666 | NC_007644 | Mta | 264732 |
| SimHC | Xylella_fastidiosa | 2679306 | 0.43 | 2880 | 10474 | NC_002488 | Xft | 160492 |
| SimHC | Silicibacter_TM1040 | 3200938 | 0.29 | 2320 | 8439 | NC_008044 | Stm | 292414 |
| SimHC | Thiobacillus_denitrificans_ATCC_25259 | 2909809 | 0.28 | 2037 | 7407 | NC_007404 | Tda | 292415 |
| SimHC | Rubrobacter_xylanophilus_DSM_9941 | 3225748 | 0.27 | 2177 | 7918 | NC_008148 | Rxd | 266117 |
| SimHC | Bacillus_cereus_B4264 | 5419036 | 0.26 | 3522 | 12809 | NC_011725 | Bcb | 405532 |
| SimHC | Burkholderia_ambifaria_MC40_6 | 3443583 | 0.26 | 2238 | 8139 | NC_010551 | Bam | 398577 |
| SimHC | Burkholderia_383 | 3694126 | 0.26 | 2401 | 8732 | NC_007510 | Bkd | 269483 |
| SimHC | Cronobacter_turicensis | 4384526 | 0.26 | 2850 | 10363 | NC_013282 | Ctu | 693216 |
| SimHC | Lactobacillus_gasseri_ATCC_33323 | 1894360 | 0.26 | 1231 | 4478 | NC_008530 | Lga | 324831 |
| SimHC | Prosthecochloris_aestuarii_DSM_271 | 2512923 | 0.26 | 1633 | 5940 | NC_011059 | Pad | 290512 |
| SimHC | Pseudoalteromonas_atlantica_T6c | 5187005 | 0.26 | 3372 | 12260 | NC_008228 | Pat | 342610 |
| SimHC | Pseudomonas_putida_F1 | 5959964 | 0.26 | 3874 | 14087 | NC_009512 | Ppf | 351746 |
| SimHC | Shewanella_MR-7 | 4792610 | 0.26 | 3115 | 11328 | NC_008322 | Smr | 60481 |
| SimHC | Alkalilimnicola_ehrlichei_MLHE-1 | 3275944 | 0.25 | 2047 | 7445 | NC_008340 | Aem | 187272 |
| SimHC | Anaeromyxobacter_dehalogenans_2CP-C | 5013479 | 0.25 | 3133 | 11394 | NC_007760 | Adh | 290397 |
| SimHC | Bradyrhizobium_BTAi1 | 8264687 | 0.25 | 5165 | 18783 | NC_009485 | Bbt | 288000 |
| SimHC | Chlorobium_chlorochromatii_CaD3 | 2572079 | 0.25 | 1608 | 5846 | NC_007514 | Ccc | 340177 |
| SimHC | Jannaschia_CCS1 | 4317977 | 0.25 | 2699 | 9814 | NC_007802 | Jcc | 290400 |
| SimHC | Kineococcus_radiotolerans_SRS30216 | 4761183 | 0.25 | 2976 | 10821 | NC_009664 | Krs | 266940 |
| SimHC | Novosphingobium_aromaticivorans_DSM_12444 | 3561584 | 0.25 | 2226 | 8095 | NC_007794 | Nad | 279238 |
| SimHC | Paracoccus_denitrificans_PD1222 | 2852282 | 0.25 | 1783 | 6482 | NC_008686 | Pdp | 318586 |
| SimHC | Pelobacter_propionicus_DSM_2379 | 4008000 | 0.25 | 2505 | 9109 | NC_008609 | Ppd | 338966 |
| SimHC | Rhodopseudomonas_palustris_BisA53 | 5505494 | 0.25 | 3441 | 12512 | NC_008435 | Rpa | 316055 |
| SimHC | Rhodopseudomonas_palustris_BisB18 | 5513844 | 0.25 | 3446 | 12531 | NC_007925 | Rpb | 316056 |
| SimHC | Rhodopseudomonas_palustris_BisB5 | 4892717 | 0.25 | 3058 | 11120 | NC_007958 | Rpc | 316057 |
| SimHC | Shewanella_frigidimarina_NCIMB_400 | 4845257 | 0.25 | 3028 | 11012 | NC_008345 | Sfn | 318167 |
| SimHC | Shewanella_ANA-3 | 4972204 | 0.25 | 3108 | 11300 | NC_008577 | San | 94122 |
| SimHC | Shewanella_W3-18-1 | 4708380 | 0.25 | 2943 | 10701 | NC_008750 | Shw | 351745 |
| SimHC | Trichodesmium_erythraeum_IMS101 | 7750108 | 0.25 | 4844 | 17614 | NC_008312 | Sww | 203124 |
| SimHC | Actinobacillus_succinogenes_130Z | 2319663 | 0.24 | 1392 | 5061 | NC_009655 | Asu | 339671 |
| SimHC | Burkholderia_vietnamiensis_G4 | 3652814 | 0.24 | 2192 | 7970 | NC_009256 | Bvg | 269482 |
| SimHC | Caldicellulosiruptor_saccharolyticus_DSM_8903 | 2970275 | 0.24 | 1782 | 6481 | NC_009437 | Csd | 351627 |
| SimHC | Chloroflexus_aurantiacus_J_10_fl | 5258541 | 0.24 | 3155 | 11473 | NC_010175 | Caj | 324602 |
| SimHC | Clostridium_beijerinckii_NCIMB_8052 | 6000632 | 0.24 | 3600 | 13092 | NC_009617 | Cbn | 290402 |
| SimHC | Deinococcus_geothermalis_DSM_11300 | 2467205 | 0.24 | 1480 | 5383 | NC_008025 | Dgd | 319795 |
| SimHC | Marinobacter_aquaeolei_VT8 | 4326849 | 0.24 | 2596 | 9440 | NC_008740 | Mav | 351348 |
| SimHC | Methanococcoides_burtonii_DSM_6242 | 2575032 | 0.24 | 1545 | 5618 | NC_007955 | Mbd | 259564 |
| SimHC | Methanospirillum_hungatei_JF-1 | 3544738 | 0.24 | 2127 | 7734 | NC_007796 | Mhj | 323259 |
| SimHC | Nitrobacter_winogradskyi_Nb-255 | 3402093 | 0.24 | 2041 | 7423 | NC_007406 | Nwn | 323098 |
| SimHC | Nitrosococcus_oceani_ATCC_19707 | 3481691 | 0.24 | 2089 | 7596 | NC_007484 | Noa | 323261 |
| SimHC | Nitrosospira_multiformis_ATCC_25196 | 3184243 | 0.24 | 1911 | 6947 | NC_007614 | Nma | 323848 |
| SimHC | Nocardioides_JS614 | 4985871 | 0.24 | 2992 | 10878 | NC_008699 | Njs | 196162 |
| SimHC | Polaromonas_JS666 | 5200264 | 0.24 | 3120 | 11346 | NC_007948 | Pjs | 296591 |
| SimHC | Prochlorococcus_marinus_NATL2A | 1842899 | 0.24 | 1106 | 4021 | NC_007335 | Pmn | 59920 |
| SimHC | Psychrobacter_cryohalolentis_K5 | 3059876 | 0.24 | 1836 | 6676 | NC_007969 | Pck | 335284 |
| SimHC | Rhodoferax_ferrireducens_T118 | 4712337 | 0.24 | 2827 | 10281 | NC_007908 | Rft | 338969 |
| SimHC | Rhodopseudomonas_palustris_HaA2 | 5331656 | 0.24 | 3199 | 11633 | NC_007778 | Rph | 316058 |
| SimHC | Saccharophagus_degradans_2-40 | 5057531 | 0.24 | 3035 | 11035 | NC_007912 | Sde | 203122 |
| SimHC | Shewanella_baltica_OS155 | 5127376 | 0.24 | 3076 | 11187 | NC_009052 | Sbo | 325240 |
| SimHC | Shewanella_putrefaciens_CN-32 | 4659220 | 0.24 | 2796 | 10166 | NC_009438 | Spn | 319224 |
| SimHC | Shewanella_loihica_PV-4/ | 4602594 | 0.24 | 2762 | 10042 | NC_009092 | Slp | 323850 |
| SimHC | Sphingopyxis_alaskensis_RB2256 | 3345170 | 0.24 | 2007 | 7299 | NC_008048 | Sar | 317655 |
| SimHC | Syntrophomonas_wolfei_Goettingen | 2936195 | 0.24 | 1762 | 6406 | NC_008346 | Swg | 335541 |
| SimHC | Arthrobacter_FB24 | 4698945 | 0.23 | 2702 | 9825 | NC_008541 | Afb | 290399 |
| SimHC | Burkholderia_cenocepacia_AU_1054 | 3294563 | 0.23 | 1894 | 6889 | NC_008060 | Bca | 331271 |
| SimHC | Burkholderia_cenocepacia_HI2424 | 3483902 | 0.23 | 2003 | 7285 | NC_008542 | Bch | 331272 |
| SimHC | Ehrlichia_chaffeensis_Arkansas | 1176248 | 0.23 | 676 | 2459 | NC_007799 | Eca | 205920 |
| SimHC | Exiguobacterium_sibiricum_255_15 | 3034136 | 0.23 | 1745 | 6344 | NC_010556 | Esi | 262543 |
| SimHC | Frankia_EAN1pec | 8982042 | 0.23 | 5165 | 18781 | NC_009921 | Fea | 298653 |
| SimHC | Frankia_CcI3 | 5433628 | 0.23 | 3124 | 11361 | NC_007777 | Fcc | 106370 |
| SimHC | Magnetococcus_MC-1 | 4719581 | 0.23 | 2714 | 9868 | NC_008576 | Mmc | 156889 |
| SimHC | Methylobacillus_flagellatus_KT | 2971517 | 0.23 | 1709 | 6213 | NC_007947 | Mfk | 265072 |
| SimHC | Nitrobacter_hamburgensis_X14 | 4406967 | 0.23 | 2534 | 9215 | NC_007964 | Mhx | 323097 |
| SimHC | Nitrosomonas_eutropha_C71 | 2661057 | 0.23 | 1530 | 5564 | NC_008344 | Nec | 335283 |
| SimHC | Pelodictyon_phaeoclathratiforme_BU_1 | 3018238 | 0.23 | 1735 | 6311 | NC_011060 | Pph | 324925 |
| SimHC | Shewanella_amazonensis_SB2B | 4306142 | 0.23 | 2476 | 9004 | NC_008700 | Sas | 326297 |
| SimHC | Streptococcus_thermophilus_LMD-9 | 1856368 | 0.23 | 1067 | 3881 | NC_008532 | Stl | 322159 |
| SimHC | Thermoanaerobacter_pseudethanolicus_ATCC_33223 | 2362816 | 0.23 | 1359 | 4940 | NC_010321 | Tpa | 340099 |
| SimHC | Thiomicrospira_crunogena_XCL-2 | 2427734 | 0.23 | 1396 | 5076 | NC_007520 | Tcx | 317025 |
| SimHC | Alkaliphilus_metalliredigens_QYMF/ | 4929566 | 0.22 | 2711 | 9859 | NC_009633 | Amq | 293826 |
| SimHC | Anabaena_variabilis_ATCC_29413 | 6365727 | 0.22 | 3501 | 12731 | NC_007413 | Ava | 240292 |
| SimHC | Azotobacter_vinelandii_DJ | 5365318 | 0.22 | 2951 | 10731 | NC_012560 | Avd | 322710 |
| SimHC | Chlorobium_limicola_DSM_245 | 2763181 | 0.22 | 1520 | 5526 | NC_010803 | Cld | 290315 |
| SimHC | Chlorobium_phaeobacteroides_DSM_266 | 3133902 | 0.22 | 1724 | 6268 | NC_008639 | Cpd | 290317 |
| SimHC | Clostridium_thermocellum_ATCC_27405 | 3843301 | 0.22 | 2114 | 7687 | NC_009012 | Cta | 203119 |
| SimHC | Desulfovibrio_desulfuricans_G20 | 3730232 | 0.22 | 2052 | 7460 | NC_007519 | Ddg | 207559 |
| SimHC | Geobacter_metallireducens_GS-15 | 3997420 | 0.22 | 2199 | 7995 | NC_007517 | Gmg | 269799 |
| SimHC | Haemophilus_somnus_129PT | 2007700 | 0.22 | 1104 | 4015 | NC_008309 | Hsp | 205914 |
| SimHC | Pediococcus_pentosaceus_ATCC_25745 | 1832387 | 0.22 | 1008 | 3665 | NC_008525 | Ppa | 278197 |
| SimHC | Pelodictyon_phaeoclathratiforme_BU_1/ | 3018238 | 0.22 | 1660 | 6036 | NC_011060 | Ppb | 324925 |
| SimHC | Prochlorococcus_marinus_MIT_9312 | 1709204 | 0.22 | 940 | 3418 | NC_007577 | Pmm | 74546 |
| SimHC | Prosthecochloris_vibrioformis_DSM_265 | 1966858 | 0.22 | 1082 | 3934 | NC_009337 | Pvd | 290318 |
| SimHC | Pseudomonas_syringae_pv_B728a | 6093698 | 0.22 | 3352 | 12187 | NC_007005 | Psp | 205918 |
| SimHC | Rhodospirillum_rubrum_ATCC_11170 | 4352825 | 0.22 | 2394 | 8706 | NC_007643 | Rra | 269796 |
| SimHC | Streptococcus_suis_98HAH33 | 2095698 | 0.22 | 1153 | 4191 | NC_009443 | Ssh | 391296 |
| SimHC | Syntrophobacter_fumaroxidans_MPOB | 4990251 | 0.22 | 2745 | 9981 | NC_008554 | Sfm | 335543 |
| SimHC | Bifidobacterium_longum_DJO10A | 2375792 | 0.21 | 1247 | 4536 | NC_010816 | Bld | 205913 |
| SimHC | Dechloromonas_aromatica_RCB | 4501104 | 0.21 | 2363 | 8593 | NC_007298 | Dar | 159087 |
| SimHC | Desulfobacterium_autotrophicum_HRM2 | 5589073 | 0.21 | 2934 | 10670 | NC_012108 | Dah | 177437 |
| SimHC | Ehrlichia_canis_Jake | 1315030 | 0.21 | 690 | 2511 | NC_007354 | Ecj | 269484 |
| SimHC | Lactobacillus_casei_ATCC_334 | 2895264 | 0.21 | 1520 | 5527 | NC_008526 | Lca | 321967 |
| SimHC | Mesorhizobium_BNC1 | 4412446 | 0.21 | 2317 | 8424 | NC_008254 | Mbn | 266779 |
| SimHC | Methanosarcina_barkeri_fusaro | 4837408 | 0.21 | 2540 | 9235 | NC_007355 | Mbf | 269797 |
| SimHC | Pelobacter_carbinolicus | 3665893 | 0.21 | 1925 | 6999 | NC_007498 | Pca | 338963 |
| SimHC | Brevibacillus_brevis_NBRC_100599 | 6296436 | 0.2 | 3148 | 11448 | NC_012491 | Bbn | 358681 |
| SimHC | Cytophaga_hutchinsonii_ATCC_33406 | 4433218 | 0.2 | 2217 | 8060 | NC_008255 | Cha | 269798 |
| SimHC | Fervidobacterium_nodosum_Rt17-B1 | 1948941 | 0.2 | 974 | 3544 | NC_009718 | Fnr | 381764 |
| SimHC | Lactococcus_lactis_cremoris_MG1363 | 2529478 | 0.2 | 1265 | 4599 | NC_009004 | Llc | 416870 |
| SimHC | Leuconostoc_mesenteroides_ATCC_8293 | 2038396 | 0.2 | 1019 | 3706 | NC_008531 | Lma | 203120 |
| SimHC | Oenococcus_oeni_PSU-1 | 1780517 | 0.2 | 890 | 3237 | NC_008528 | Oop | 203123 |
| SimHC | Pseudomonas_fluorescens_Pf0_1 | 6438405 | 0.2 | 3219 | 11706 | NC_007492 | Pfp | 205922 |
| SimHC | Synechococcus_PCC_7002 | 3008047 | 0.2 | 1504 | 5469 | NC_010475 | Spc | 32049 |
| SimHC | Thermobifida_fusca_YX | 3642249 | 0.2 | 1821 | 6622 | NC_007333 | Tfy | 269800 |
| SimHC | Thiomicrospira_denitrificans_ATCC_33889 | 2201561 | 0.2 | 1101 | 4003 | NC_007575 | Tdt | 326298 |
| SimHC | Burkholderia_xenovorans_LB400 | 4895836 | 0.19 | 2326 | 8456 | NC_007951 | Bxl | 266265 |
| SimHC | Lactobacillus_brevis_ATCC_367 | 2291220 | 0.19 | 1088 | 3958 | NC_008497 | Lba | 387344 |
| SimHC | Chromohalobacter_salexigens_DSM_3043 | 3696649 | 0.18 | 1663 | 6049 | NC_007963 | Css | 290398 |
| SimHC | Lactobacillus_delbrueckii_bulgaricus_ATCC_BAA-365 | 1856951 | 0.18 | 836 | 3039 | NC_008529 | Ldb | 321956 |
| SimHC | Psychrobacter_arcticum_273-4 | 2650701 | 0.18 | 1193 | 4338 | NC_007204 | Par | 259536 |
| SimHC | Rhodobacter_sphaeroides_KD131 | 3152792 | 0.18 | 1419 | 5159 | NC_011963 | Rsk | 557760 |
| SimHC | Enterococcus_faecalis_V583 | 3218031 | 0.17 | 1368 | 4973 | NC_004668 | Efv | 226185 |
| SimHC | Xylella_fastidiosa_M12 | 2475130 | 0.09 | 557 | 2025 | NC_010513 | Xfm | 405440 |
